# Genome-wide identification and characterization of aquaporin gene family in *Beta vulgaris*

**DOI:** 10.7717/peerj.3747

**Published:** 2017-09-19

**Authors:** Weilong Kong, Shaozong Yang, Yulu Wang, Mohammed Bendahmane, Xiaopeng Fu

**Affiliations:** 1 College of Horticulture and Forestry Sciences, Huazhong Agricultural University, Key Laboratory of Horticultural Plant Biology, Ministry of Education, Wuhan, Hubei, China; 2 INRA-CNRS-Lyon1-ENS, Laboratoire Reproduction et Developpement des Plantes, Ecole Normale Supérieure Lyon, France

**Keywords:** Gene structure, Expression profile, *Beta vulgaris*, Aquaporins, Abiotic stress

## Abstract

Aquaporins (AQPs) are essential channel proteins that execute multi-functions throughout plant growth and development, including water transport, uncharged solutes uptake, stress response, and so on. Here, we report the first genome-wide identification and characterization *AQP* (*BvAQP*) genes in sugar beet (*Beta vulgaris*)*,* an important crop widely cultivated for feed, for sugar production and for bioethanol production. Twenty-eight sugar beet *AQPs* (*BvAQPs*) were identified and assigned into five subfamilies based on phylogenetic analyses: seven of plasma membrane (PIPs), eight of tonoplast (TIPs), nine of NOD26-like (NIPs), three of small basic (SIPs), and one of x-intrinsic proteins (XIPs). *BvAQP* genes unevenly mapped on all chromosomes, except on chromosome 4. Gene structure and motifs analyses revealed that *BvAQP* have conserved exon-intron organization and that they exhibit conserved motifs within each subfamily. Prediction of BvAQPs functions, based on key protein domains conservation, showed a remarkable difference in substrate specificity among the five subfamilies. Analyses of *BvAQPs* expression, by mean of RNA-seq, in different plant organs and in response to various abiotic stresses revealed that they were ubiquitously expressed and that their expression was induced by heat and salt stresses. These results provide a reference base to address further the function of sugar beet aquaporins and to explore future applications for plants growth and development improvements as well as in response to environmental stresses.

## Introduction

AQPs are known to facilitate water transport and other small nutrients through cell membranes (Maurel et al., 2009; Maurel et al., 2008). Since the discovery of the first aquaporin (AQP1) in mammals, AQPs were identified in many microorganisms, plants and animals (Gomes et al., 2009).

Plant aquaporin families are complex, and are composed of a large number of genes. For example, there are 35 AQPs in *Arabidopsis thaliana*, 31 in *Zea mays*, 34 in *Oryza sativa*, 55 in *Populus trichocarpa* and 66 AQPs in *Glycine max* (Johanson & Kjellbom, 2001; Nguyen, Moon & Jung, 2013; Chaumont et al., 2001; Gupta & R, 2009; Zhang et al., 2013). In plants AQPs play major roles in water and solute transport, in maintaining water homeostasis and in the response to environment stresses. AQPs roles in glycerol, urea, boric acid, silicic acid, H_2_O_2_, NH_3_ and CO_2_ transport through cell membranes were reported to be important for cytoplasm homeostasis, seed germination, embolism recovery, petal and leaf movement, guard cells closure, fruit ripening and maintenance of cell turgor under various stresses (Fitzpatrick & Reid, 2009; Maurel et al., 2009; Maurel et al., 2008; Mitani-Ueno et al., 2011; Heinen & Chaumont, 2009; Maurel et al., 2008; Prado & Maurel, 2013; Uehlein & Kaldenhoff, 2008; Wudick & Maurel, 2009).

To date, AQPs are recognized into seven subfamilies: PIPs, TIPs, NIPs, SIPs, XIPs, GIPs, and HIPs (Anderberg, Kjellbom & Johanson, 2012; Danielson & Johanson, 2008). Green plants usually contain four subfamilies: PIPs, TIPs, NIPs, and SIPs. However, members of XIPs subfamily were also found in some dicots, such as *Solanum lycopersicum* (Venkatesh, Yu & Park, 2013), *Populus trichocarpa* (Gupta & Sankararamakrishnan, 2009) and *Glycine max* (Cheng et al., 2013), but were absent in Brassicaceae and monocots (Danielson & Johanson, 2008). GIPs and HIPs subfamilies were reported in moss (*Physcomitrella patens*) and fern (*Selaginella moellendorffii*).

AQPs are highly conserved in all living organisms, consisting of six transmembrane domains (TM1–TM6) connected by five loops (LA-LE). NPA motifs, the ar/R selectivity and Froger’s position are critical for AQPs functions. Two NPA motifs (Asn-Pro-Ala) are located on LB and LE, forming a central aqueous pore in the middle of the lipid bilayer involved in proton exclusion and substrate selectivity (Bansal & Sankararamakrishnan, 2007). The ar/R selectivity is formed by four resides from TM2 (H2), TM5 (H5), LE (LE1 and LE2) and acts as a size-exclusion barrier (Hove & Bhave, 2011; Mitani-Ueno et al., 2011). Froger’s position consists of five conserved resides (P1–P5) and discriminations between AQP-type and GIP-type AQPs (Froger et al., 1998). Nine specificity-determining positions for non-aqua substrates (i.e., urea, boric, acid, silicic, ammonia, carbon dioxide and hydrogen peroxide (H_2_O_2_)) were also proposed based on a comprehensive analysis of functionally characterized AQPs (Hove & Bhave, 2011).

Sugar beet belongs to Caryophyllales, which lays on basal taxa of core dicots. It is an important crop in temperate climates region and provides nearly 30% of the world’s annual sugar production (Dohm et al., 2014). It is also used as a source for animal feed and for bioethanol production.

To date, little is known about AQPs in sugar beet and in Caryophyllales. So far, only information on 26 AQPs, grouped in five subfamilies (eight PIPs, 11 TIPs, four NIPs, two SIPs and one XIP), was reported in carnation (Morita et al., 2017). More information on AQPs in Caryophyllales plants is therefore required to help understand their function and evolution.

Here we used the available high-quality genome sequence (Dohm et al., 2014) and RNA-seq datasets (Minoche et al., 2015) of sugar beet to identify and characterize the expression of BvAQPs. We report the distribution of BvAQPs on chromosomes, phylogeny analysis, gene structure, subcellular location, conserved resides and conserved elements. We propose putative functions of sugar beet AQPs based on their detailed genes expression patterns analysis by means of RNA-seq.

## Methods

### Identification and distribution of AQP genes in sugar beet

BvAQPs were identified by HMM (Hidden Markov Model) and BLAST homology search. The sugar beet predicted proteome was collected using the sugar beet genome RefBeet-1.2 (http://bvseq.molgen.mpg.de/Genome/Download/index.shtml). The Hidden Markov model (HMM) of the MIP domain (PF00230) was downloaded from the Sanger database (http://pfam.xfam.org/family/PF00230). PF00230 was then used to query the predicted Sugar beet proteome using HMMER 3.0 software (http://hmmer.org/). 35 Arabidopsis AtAQPs were download from TAIR Database (https://www.arabidopsis.org/browse/genefamily/Aquaporins.jsp) and then used to search BvAQPs with BLASTp tool using NCBI sugar beet genome (https://www.ncbi.nlm.nih.gov/genome/?term=Beta+vulgaris) and sugar beet genome (http://www.genomforschung.uni-bielefeld.de/en/projects/annobeet) (Baranwal, Negi & Khurana, 2016) with cut-off *E*-value of e^−5^. All no-redundant gene sequences were analyzed by SMART (http://smart.embl-heidelberg.de/) and Pfam (http://pfam.xfam.org/search/sequence). Sequences encoding complete MIP domain and two NPA motifs were considered as putative AQP genes.

Additionally, the molecular weight (MW) and isoelectric point (PI) of BvAQPs were calculated by ExPASy (http://www.expasy.org/); transmembrane helical domains (TMHs) were assessed by TMHMM Sever v.2.0 (http://www.cbs.dtu.dk/services/TMHMM/); the subcellular localization of BvAQPs were predicted using Plant-mPLoc (http://www.csbio.sjtu.edu.cn/bioinf/plant-multi/) and WolF PSORT (http://www.genscript.com/wolf-psort.html). The position of the *AQP* genes on the sugar beet chromosomes were identified based on position information from the sugar beet genome database and the distribution graph of *AQP* genes was drawn by MapInspect software (http://mapinspect.software.informer.com/).

### Phylogenetic analyses and sequence alignments

BvAQPs were aligned with AtAQPs from Arabidopsis (https://www.arabidopsis.org/) DcAQPs from *Dianthus caryophyllus* (Morita et al., 2017; http://carnation.kazusa.or.jp/blast.html), by using Clustal W. Phylogenetic tree was built by MEGA6.0 (http://www.megasoftware.net/history.php) using the neighbor-joining (NJ) method, with 1,000 times bootstrap replicates. BvAQPs were named based on their sequence homology and phylogenetic analyses. Greek letters (α, β) were used to denote the transcripts derived from the same gene.

BvAQPs and two *S. tuberosum* AQPs (Venkatesh, Yu & Park, 2013) were aligned by DNAMAN (http://dnaman.software.informer.com/) with default parameters. Two NPA motifs, ar/R selectivity filter and Froger’s position were inferred from the multiple sequence alignment result from DNAMAN. Specificity-determining positions (SDP1-SDP9) from alignments with the structure resolved *Spinacia oleracea* PIP2;1 and functionally characterized AQPs as being collected by Hove and Bhave (Hove & Bhave, 2011).

### Exon-intron structure, tandem duplication events and conserved motifs distribution

The exon-intron structure was performed by GSDS 2.0 (http://gsds.cbi.pku.edu.cn/) based on genes coding sequences and on the annotated genome. Tandem duplication events were analyzed in *BvAQP* genes (Gu et al., 2002; He et al., 2012). Conserved motifs of BvAQPs were analyzed by MEME suit (http://meme-suite.org/), and the parameters were set as follows: maximum number is 10, other default parameters.

### Expression analysis of sugar beet AQP genes

RNA-seq data (SRX287608–SRX287615) were collected from NCBI and used to analyse the expression profiles of AQP genes in different organs and tissues (seedling, root, leaf, inflorescence and seed). Salt and heat tolerance is largely dependent on the plant ability to maintain optimal water status in leaves. The adjustment of water relation under salinity involves changes in the transcriptional activity of genes encoding AQPs. Expression variations of *BvAQPs* in young leaf under salt or heat stress, were analyzed using RNA-seq data (SRX647324; SRX647712; SRX647714) (Minoche et al., 2015).

HISAT2 was used to align raw reads to the reference genome, and StringTie was used to calculate gene expression (Stracke et al., 2014). The heat map for tissue-specific expression profile was generated based on the Log${}_{2}^{\mathrm{RPKM}}$ values for each gene in all the tissue samples using R package (Gentleman et al., 2004). Stress inducible profile of *BvAQP* genes in young leaf, RPKM values were normalized to untreated controls, and expression fold changes in genes were shown in terms of Log${}_{2}^{\mathrm{fold}}$ (Venkatesh, Yu & Park, 2013).

## Results

### Identification, classification and properties of *BvAQP* genes in sugar beet

**Figure 1:**
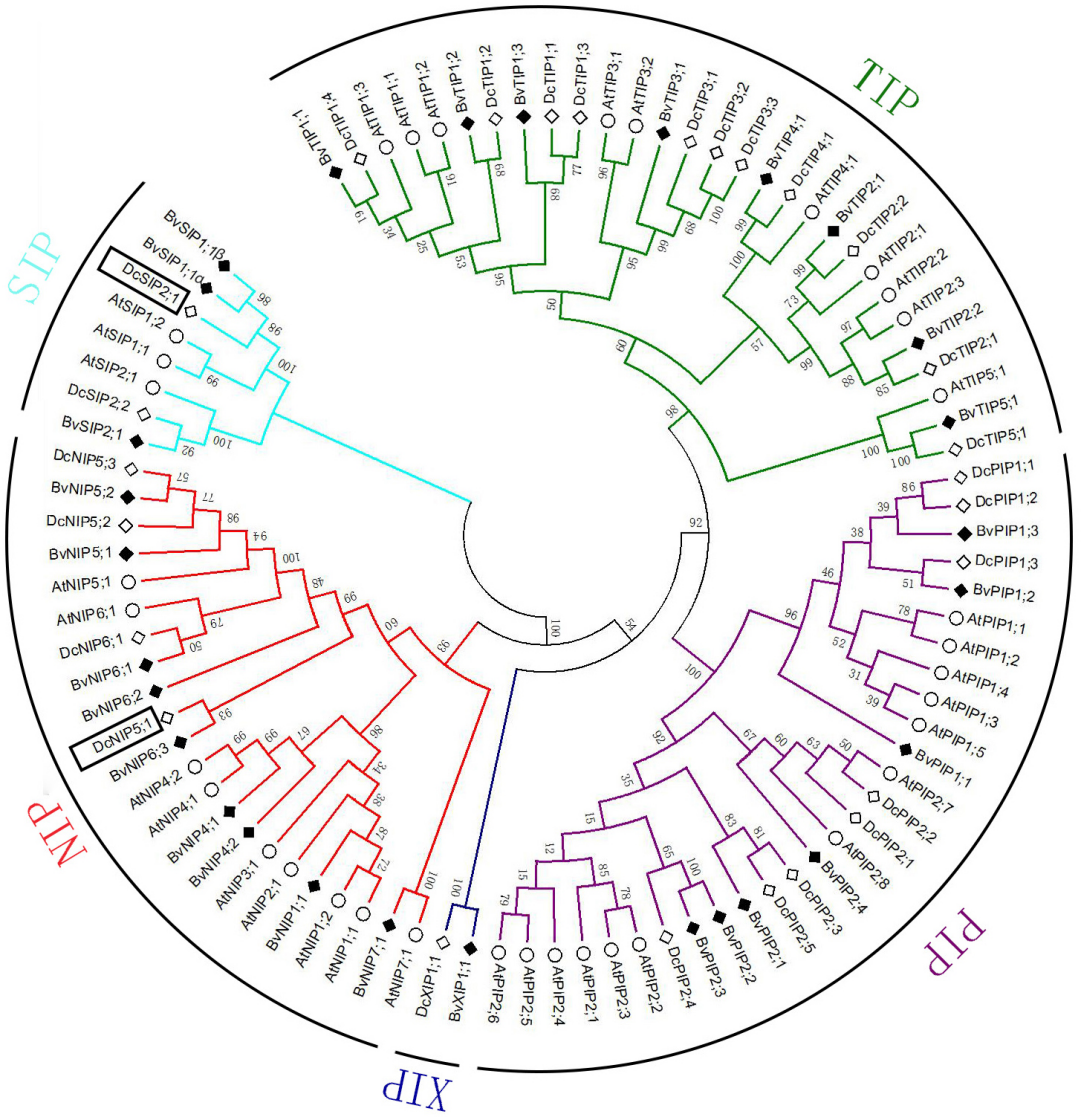
Multiple alignments and phylogenetic analysis of BvAQPs *A. thaliana* AtAQPs and *D. caryophyllus* DcAQPs. Multiple alignments were performed using the default parameter of Clustal W. Phylogenetic dendrogram was generated by MEGA 6 using neighbor-joining (NJ) method with 1,000 bootstrap replicates.

In total 28 non-redundant genes were assigned as putative *AQP*s. The predicted protein sequence of all AQPs ranged from 236 to 327 amino acids (Table 1). Sequence cluster analysis of AQPs from *A. thaliana*, *D. caryophyllus* and sugar beet permitted to group them into five subfamilies: seven PIPs, eight TIPs, nine NIPs, three SIPs and one XIPs (Fig. 1). PIPs subfamily was further divided into three PIP1s and four PIP2s subgroups. The TIPs subfamily included five subgroups (three TIP1s, two TIP2s, one TIP3, one TIP4, one TIP5). NIPs subfamily was dived into five subgroups (one NIP1, two NIP4s, two NIP5s, three NIP6s, one NIP7). SIPs subfamily divided into two subgroups (two SIP1s, one SIP2). Only one member (XIP1) composed XIPs subfamily. A pair of transcripts were found in SIP1 subgroup, named BvSIP1;1 α and SIP1;1 β.

**Table 1:** Identification of *BvAQP* genes using sugar beet genome data.

Family	Gene	Gene ID	Gene code	Protein length (aa)	MW (kDa)	pI	TMHs	Plant-mPLoc	WoLF PSORT
PIP	BvPIP1;1	fpur	Bv1g004510_fpur.t1	289	31.1	8.84	5	plas	cyto
	BvPIP1;2	shpz	Bv1g004520_shpz.t1	285	30.67	9.05	6	plas	plas
	BvPIP1;3	gqok	Bv2g024120_gqok.t1	286	30.74	9.14	6	plas	plas
	BvPIP2;1	cqnr	Bv7g163390_cqnr.t1	284	30.32	8.31	6	plas	plas
	BvPIP2;2	ixem	Bv9g210030_ixem.t1	288	31.09	7.08	6	plas	plas
	BvPIP2;3	yige	Bv9g210020_yige.t1 (XP_010689549.1)^a^	274	29.64	8.97	6	plas	plas
	BvPIP2;4	reke	Bv9g216070_reke.t1	281	30.13	8.84	6	plas	plas
TIP	BvTIP1;1	kzkq	Bv7ug180930_kzkq.t1	254	26.06	5.38	6	vacu	plas
	BvTIP1;2	iuuk	Bv2g037380_iuuk.t1	252	26.3	5.92	6	vacu	vacu
	BvTIP1;3	ynzf	Bv7g176430_ynzf.t1	248	25.46	5.13	6	vacu	vacu
	BvTIP2;1	dkzm	Bv9g223310_dkzm.t1	247	25.26	5.6	6	vacu	plas
	BvTIP2;2	xunf	Bv5g104980_xunf.t1	249	25.09	4.7	7	vacu	vacu
	BvTIP3;1	dreg	Bv8g190600_dreg.t1	257	27.13	7.07	5	vacu	nucl/mito/vacu
	BvTIP4;1	ydno	Bv2g032200_ydno.t1	247	26.1	6.57	6	vacu	cyto/vacu
	BvTIP5;1	gghp	Bv3ug068240_gghp.t1	255	26.54	8.47	6	plas	chlo
NIP	BvNIP1;1	ughi	Bv8ug202570_ughi.t1	292	30.87	8.91	6	plas	plas
	BvNIP4;1	aejh	Bv2g027680_aejh.t1	273	29.3	8.87	6	plas	vacu
	BvNIP4;2	xash	Bv2g027660_xash.t1	299	32.62	8.27	6	plas	plas
	BvNIP5;1	gkiq	Bv6TE021760_gkiq.t1 (XP_010680949.1)^a^	261	27.61	8.96	6	plas	vacu
	BvNIP5;2	oani	Bv6g139140_oani.t1	298	30.9	8.73	6	plas	plas
	BvNIP6;1	hmzo	Bv9g225280_hmzo.t1	306	31.75	7.66	6	plas	plas
	BvNIP6;2	zkgo	Bv5g108450_zkgo.t1 (XP_010677474.1)^a^	266	27.46	7.82	6	plas	plas
	BvNIP6;3	jecw	Bv5g108440_jecw.t1 (XP_010677699.1)^a^	327	35.08	9.03	6	plas	plas
	BvNIP7;1	kqew	Bv3ug070540_kqew.t1	289	30.72	7.13	7	plas	plas
SIP	BvSIP1;1 α	zywx	Bv2g035790_zywx.t1 (XP_010669579.1)^a^	250	26.46	9.46	5	plas	plas
	BvSIP1;1 β	fzwq	Bv2g035780_fzwq.t1	247	26.39	9.74	6	plas/vacu	vacu
	BvSIP2;1	qzqg	Bv3g064810_qzqg.t1 (XP_010673209.1)^a^	236	26.02	9.56	5	plas	vacu
XIP	BvXIP1;1	iwpe	Bv9g217040_iwpe.t1	312	34.13	8.38	7	plas	plas

**Notes.**

Note1: Best possible cell localization prediction by the Plant-mPLoc and WoLF PSORT tool (Chlo: chloroplast; Cyto: cytosol; Cysk: cytoskeleton; E.R: endoplasmic reticulum; Extr: extracellular; Golg: Golgi apparatus; Lyso: lysosome; Mito: mitochondria; Nucl: nuclear; Pero: peroxisome; Plas: plasma membrane; Vacu: vacuolar membrane).

a sequences were splicing errors in AnnoBeet, and were corrected by NCBI Beta vulgaris (ID 221)—Genome. These were verified by PCR amplification.

Bioinformatics analysis revealed that MW of BvAQPs ranged from 25.09 to 35.08 kDa with a pI between 4.7 and 9.74 (Table 1). TIPs and SIPs were smaller (<27 kDa) than PIPs, NIPs and XIPs. TIPs were acidic while the other subfamilies were alkaline. The great majority of AQPs were predicted have six TMHs, while BvPIP1;1, BvTIP3;1, BvSIP1;1 α and BvSIP2;1 had only five TMHs and BvTIP2;2, BvNIP7;1 and BvXIP1;1 had seven TMHs.

### Genes mapping on the sugar beet chromosomes, gene duplications, gene structure and alternative splicing

**Figure 2:**
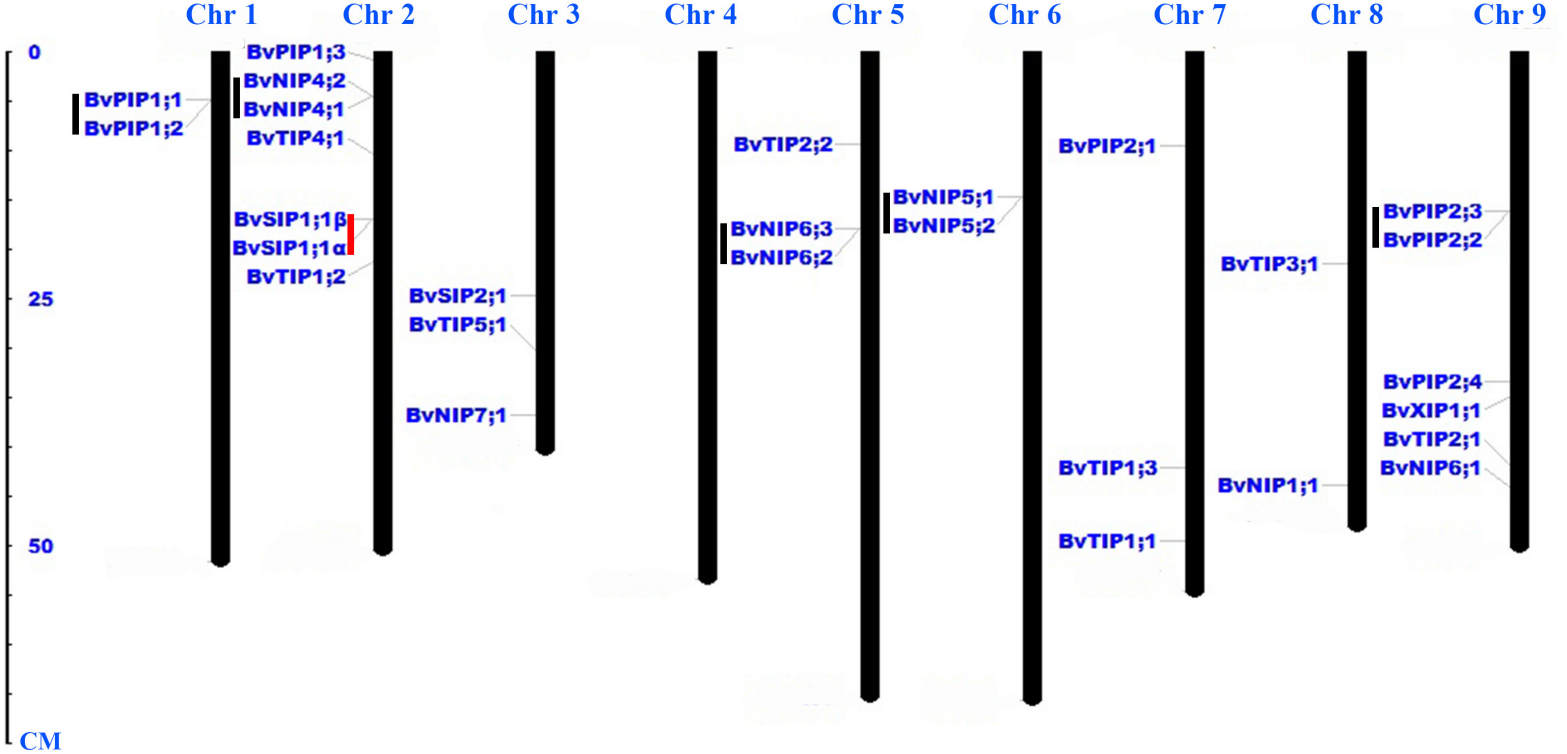
Distribution of *BvAQP* genes on the nine *B. vulgaris* chromosomes. Note: Black lines represent tandem gene duplications. Red lines represent the existence of different transcripts from single gene.

Twenty-eight *AQP* genes were unevenly mapped on eight chromosomes (Fig. 2). Seven *BvAQP* genes (25%) mapped on chromosome (Chr) 2, 6 *BvAQP* genes (21%) mapped on Chr9. Chr3, Chr5 and Chr7 each had three *BvAQP* genes (10%) and Chr1, Chr6 and Chr8 each had two *BvAQP* genes. No putative AQP was found on Chr4. Tandem duplication events were found on Chr1, Chr2, Chr5, Chr6 and Chr8 (i.e., *BvPIP1;1* and *BvPIP1;2*, *BvNIP4;1* and Bv*NIP4;2*, *BvNIP6;2* and *BvNIP6;3*, *BvNIP5;1* and *BvNIP5;2*, *BvPIP2;2* and *BvPIP2;3*).

**Figure 3:**
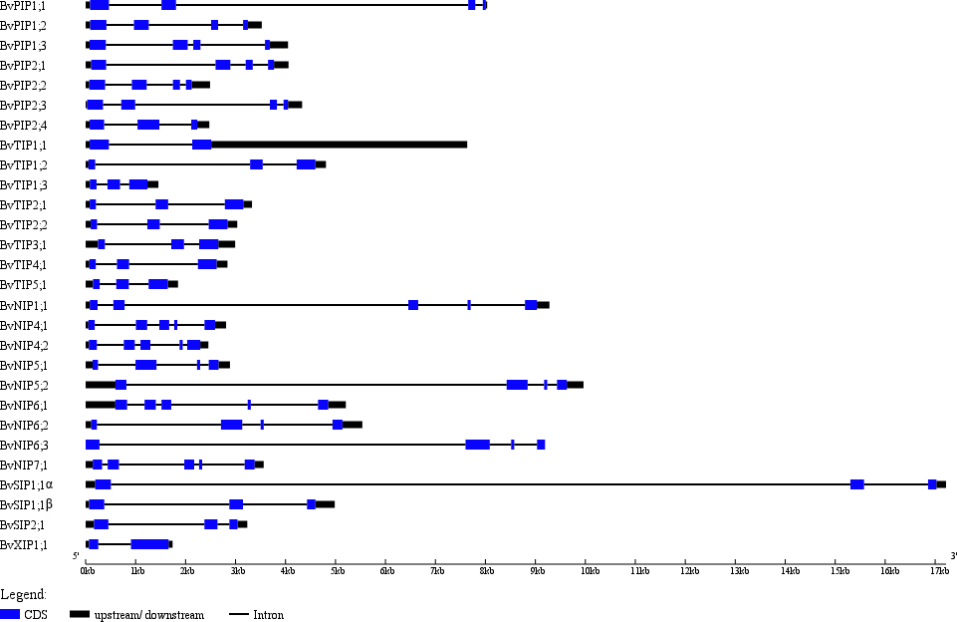
*BvAQP* **genes structure analysis**.

The exon-intron structures play crucial roles during plant evolution. The sugar beet AQPs exon-intron structures are showed in Fig. 3. Most PIP subfamily genes have four exons, while *BvPIP1;3* had 5 and *BvPIP2;4* had three. TIP subfamily genes had three exons; specifically, the third exon of *BvTIP1;1* had a noncoding exon and codes a long 5′ translated region (5′ UTR). NIP subfamily genes had five exons with the exception *BvNIP5;2*, *BvNIP6;2* and *BvNIP6;3* had four exons. Most SIP subfamily genes have three exons but *BvXIP1;1* only had two exons, which were similar to situations reported in common bean, tomato, potato, and so on. (Ariani & Gepts, 2015; Reuscher et al., 2013a; Venkatesh, Yu & Park, 2013). These results suggested that BvAQP gene structure is globally conserved in sugar beet. The conserved exon-intron structure provided an additional proof to support the classification results (Fig. 1).

**Figure 4:**
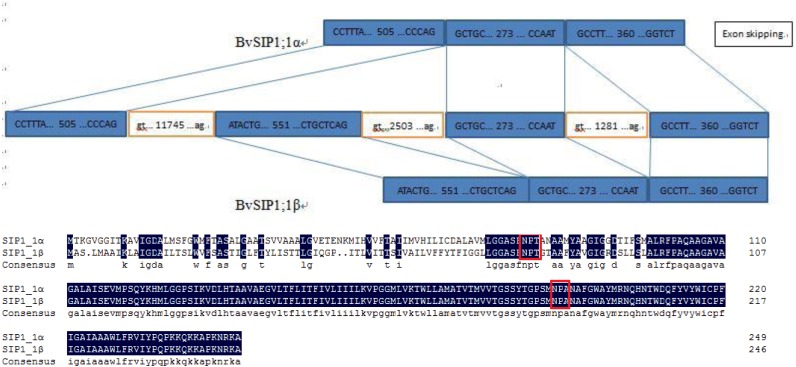
Alternative splicing of *SIP1;1* generate two different transcripts: *SIP1;1α* and *SIP1;1β*.

To seek further insights into the gene structure, the splicing pattern of sugar beet *BvAQP* pre-mRNA sequence were analyzed. The splicing analysis revealed that *BvSIP1;1 α* is the result of second exon skipping (Fig. 4). The amino acid sequence of *BvSIP1;1α* and *BvSIP1;1β* shows a 74.8% similarity.

### Conserved resides and conserved motifs distribution

**Figure 5:**
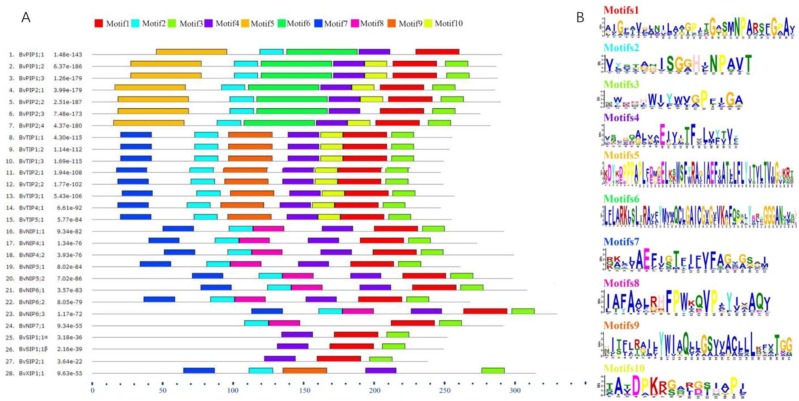
BvAQPs protein motifs identified by MEME. Using the complete amino acid sequences of BvAQPs. Combined *p*-values are indicated in (A) and different motifs were shown by different colors and numbered from 1 to 10 in (B).

To further understand the possible physiological role and substrate specificity of BvAQPs, they were aligned and TMHs and conserved resides (NPA motifs, ar/R selectivity filter and Froger’s position) were analyzed (Table 2). The data revealed that all BvAQPs had six characteristic TMHs for AQPs, required for transport function (Fig. S1). However, BvPIP1;1 showed partial loss of TM6. The resulting missing protein domains may cause dominant negative effect on protein functions, although this need to be confirmed. All PIPs and TIPs showed the dual typical NPA motifs, but some members of NIPs such as BvNIP5;1, BvNIP5;2 and BvNIP6;1 showed that the Alanine (A) in the third residue of the second NPA motif was replace d by a Valine (V). All SIPs and XIPs showed various third residues in the first NPA motif, in which Alanine (A) was replaced by Threonine (T) or by a Leucine (L). PIPs’ ar/R selectivity filter and Froger’s position showed an apparent family-specificity compared to NPA motifs, with ar/R filter configuration typical for water-transporting AQPs (F-H-T-R) and Q/M-S-A-F-W residues in Froger’s position. TIPs contained the H-I-A/G-V/R residues in the ar/R selectivity filter and T-S/A-A-Y-W residues in Froger’s position, but BvTIP5;1 showed that the N-V-G-Y in the ar/R selectivity filter was different from other TIPs and formed a single-gene clade with the TIPs. In NIPs, the ar/R selectivity filter and Froger’s position had multiple types. BvNIP1;1, BvNIP4;1 and BvNIP4;2 showed W-V-A-R in the ar/R selectivity filter and F/L-S-A-Y-L/I in the Froger’s position. BvNIP5;1 and BvNIP5;2 showed A-I-G/A-R in the ar/R selectivity filter and F-T-A-Y-M in the Froger’s position. BvNIP6;1, BvNIP6;2 and BvNIP6;3 showed S-I-G/A-R in the ar/R selectivity filter and Y-T-A-Y-F/M/L in the Froger’s position. BvNIP7;1 showed A-V-G-R in the ar/R selectivity filter and F-S-A-Y-F in the Froger’s position. SIP1s and SIP2s showed distinct difference in the ar/R selectivity filter and the Froger’s position. BvSIP1;1 α and BvSIP1;1 β showed I/V-V-P-N ar/R selectivity filter and M-A-A-Y-W in the Froger’s position, but BvSIP2;1 showed S-N-G-S ar/R selectivity filter and F-V-A-Y-W in the Froger’s position. BvXIP1;1 showed V-S-A-R ar/R selectivity filter and F-C-A-F-W in the Froger’s position. To explore further the diversity in each group, the conserved motifs were predicted (Fig. 5). Motifs 5 and 6 appeared specifically in PIPs; Motif 8, in NIPs; Motif 9 in TIPs and XIPs.

**Table 2:** Conserved dual NPA motifs, ar/R (H2, H5, LE1 and LE2), Froger’s positions (P1–P5) analysis of BvAQPs.

Subfamily	Gene	NPA		Ar/R selectivity filter				Froger’s position				
		LB	LE	H2	H5	LE1	LE2	P1	P2	P3	P4	P5
PIP	BvPIP1;1	NPA	NPA	F	H	T	R	Q	S	A	F	W
	BvPIP1;2	NPA	NPA	F	H	T	R	Q	S	A	*	*
	BvPIP1;3	NPA	NPA	F	H	T	R	Q	S	A	F	W
	BvPIP2;1	NPA	NPA	F	H	T	R	Q	S	A	F	W
	BvPIP2;2	NPA	NPA	F	H	T	R	Q	S	A	F	W
	BvPIP2;3	NPA	NPA	F	H	T	R	Q	S	A	F	W
	BvPIP2;4	NPA	NPA	F	H	T	R	M	S	A	F	W
TIP	BvTIP1;1	NPA	NPA	H	I	A	V	T	S	A	Y	W
	BvTIP1;2	NPA	NPA	H	I	A	V	T	S	A	Y	W
	BvTIP1;3	NPA	NPA	H	I	A	V	T	S	A	Y	W
	BvTIP2;1	NPA	NPA	H	I	G	R	T	S	A	Y	W
	BvTIP2;2	NPA	NPA	H	I	G	R	T	S	A	Y	W
	BvTIP3;1	NPA	NPA	H	I	A	R	T	A	A	Y	W
	BvTIP4;1	NPA	NPA	H	I	A	R	T	S	A	Y	W
	BvTIP5;1	NPA	NPA	N	V	G	Y	T	S	A	Y	W
NIP	BvNIP1;1	NPA	NPA	W	V	A	R	F	S	A	Y	L
	BvNIP4;1	NPA	NPA	W	V	A	R	F	S	A	Y	I
	BvNIP4;2	NPS	NPA	W	A	A	R	L	S	A	Y	I
	BvNIP5;1	NPS	NPV	A	I	G	R	F	T	A	Y	M
	BvNIP5;2	NPS	NPV	A	I	A	R	F	T	A	Y	M
	BvNIP6;1	NPS	NPV	S	I	G	R	F	T	A	Y	F
	BvNIP6;2	NPA	NPA	S	I	G	R	Y	T	A	Y	M
	BvNIP6;3	NPA	NPA	S	I	A	R	Y	T	A	Y	L
	BvNIP7;1	NPA	NPA	A	V	G	R	F	S	A	Y	F
SIP	BvSIP1;1α	NPT	NPA	I	V	P	N	M	A	A	Y	W
	BvSIP1;1β	NPT	NPA	V	V	P	N	M	A	A	Y	W
	BvSIP2;1	NPL	NPA	S	N	G	S	F	V	A	Y	W
XIP	BvXIP1;1	NPT	NPA	V	S	A	R	F	C	A	F	W

### Subcellular localization

Plant-mPLoc prediction was used to predict the BvAQPs subcellular localization (Table 1). PIPs, NIPs, TIPs and SIPs (except BvSIP1;1 β) were predicted to localize to plasma membrane and all TIPs were also predicted to localize on vacuolar membrane. However, subcellular localizations predicted by WoLF PSORT were diverse and not always in agreement with that predicted by Plant-mPLoc (Table 1). For example, most PIPs were predicted to localize on plasma membrane. TIPs were predicted to localize on vacuolar membrane, plasma membrane as well as in the nucleus, mitochondria, cytosol and chloroplast. NIPs, SIPs and XIPs were predicted to localize on vacuolar membrane and plasma membrane. Although these predications represent a starting base, more experiments, i.e., with tagged proteins, are will be useful better characterize the subcellular localization of BvAQPs.

**Figure 6:**
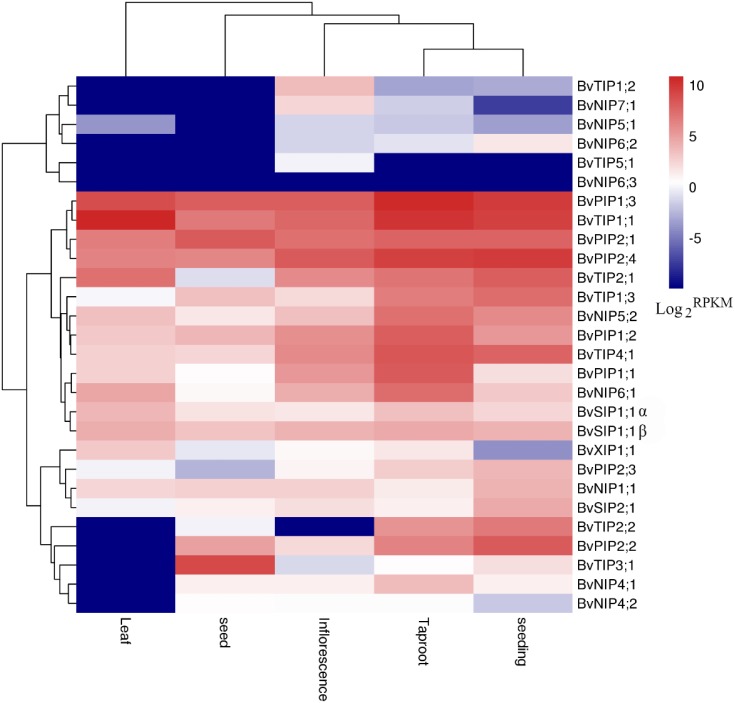
Expression profiles of the 28 *BvAQP* genes in different plant organs and tissues.

**Figure 7:**
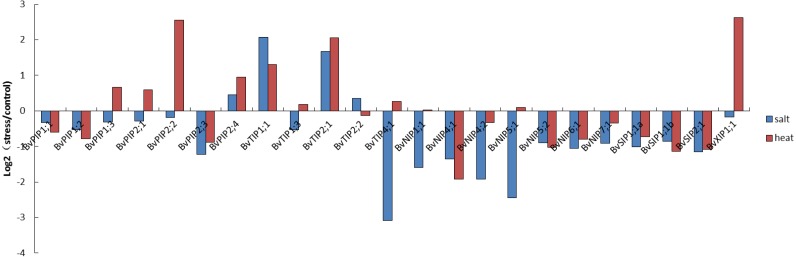
Expression pattern of the 23 *BvAQP* genes in young leaf under abiotic stress.

### Expression analysis of sugar beet AQP genes in different organs and in response to salt & heat stress

We used the available RNA-seq data from different organs and young leaf under heat, salt stress treatment of *B. vualgaris* (Dohm et al., 2014; Minoche et al., 2015) to evalute tha expression of *BvAQPs*. 27 *BvAQP* genes (96%) were expressed in at least one tissue (Fig. 6, Table S1) and one *BvAQP* gene (*NIP6;3*) did not show any expression in all tested plant organs. Eighteen *BvAQP* genes (64%) expressed in all organs, including six *PIP*, four *TIP*, four *NIP*, three *SIP* and one *XIP*. Twenty-six *BvAQP* genes (92%) expressed in taproot, with *BvPIP1;3*, *BvTIP1;1* and *BvPIP2;4* exhibiting the highest expression levels. Twenty-six *AQP* genes (92%) were expressed in inflorescence, among which *BvPIP2;4*, *BvPIP1;3* and *BvTIP1;1* exhibiting the highest expression levels. Twenty-two *AQP* genes (79%) were expressed in seed (with *BvTIP3;1*, *BvPIP2;1*, *BvPIP1;3* and *BvTIP1;1* showing the highest expression levels). Only 18 *AQP* genes (64%) were expressed in leaf (*BvTIP1;1*, *BvPIP1;3* and *BvTIP2;1* showed the highest expression levels). Most genes were expressed constitutively in the tested tissues, while one gene (*BvTIP5;1*) showed tissue-specific expression in the inflorescence. In addition, the transcripts of *BvTIP1;2* and *BvNIP7;1* were also shown to be relatively abundant in inflorescence and *BvTIP2;2* was highly expressed in taproot and seeding.

The expression patterns of sugar beet AQP genes were found to fluctuate under different stress conditions. Eleven *AQP* genes (44%) expression were down-regulated both under salt and heated stress. These include *BvPIP1;1*, *BvPIP1;2*, *BvPIP2;3*, *BvNIP4;1*, *BvNIP4;2*, *BvNIP5;2*, *BvNIP6;1*, *BvNIP7;1*, *BvSIP1;1 α*, *BvSIP1;1 β* and *BvSIP2;1* (Fig. 7). In contrast, the expression of *BvPIP2;4*, *BvTIP1;1* and *BvTIP2;1* (12%) were up-regulated both under salt and heated stress. Interestingly, nine *AQP* genes showed different expression in response to salt or heat treatments. Eight among these *AQP* genes (32%; *BvPIP1;3*, *BvPIP2;1*, *BvPIP2;2*, *BvTIP1;3*, *BvTIP4;1*, *BvNIP1;1*, *BvNIP5;1* and *BvXIP1;* 1) were up-regulated under heat treatment but down-regulated under salt treatment. Notably, members of *PIP* genes and *XIP* genes (*BvPIP1;3*, *BvPIP2;1*, *BvPIP2;2*, *BvXIP1;1*) were highly up-regulated under heat stress but slightly down-regulated under salt stress. Only *BvTIP2;2* (4%) was up-regulated under salt but down-regulated under heat stress.

Interestingly, *BvNIP6;3* wasn’t expressed in all organs, neither in young leaf under salt and heat stress (Fig. 6; Table S1), and BvNIP6;3 formed a single-gene clade within the NIP6s, with the highest MV (35.08kDa) and longest protein length (Table 1). In addition, gene duplication analysis revealed *BvNIP6;3* and *BvNIP6;2* were tandem duplication (Fig. 2) and suggested that *BvNIP6;3* was originated from *BvNIP6;2* and was pseudo-genes.

Other genes, such as *BvTIP1;2*, *BvTIP3;1*, *BvNIP6;2* and *BvNIP6;3* were not expressed in non-treated or in stressed leafs. *BvTIP5;1* was not expressed in control or heat stressed young leafs, but showed only very expression in young leaf under salt stress (Table S1).

## Discussion

Many studies have confirmed that *AQP* genes were involved in plant water transport, in regulating growth and development and that they are widely distributed in animals and plants. Most functional studies of *AQP* genes were mainly conducted using model plants, such as *A. thaliana*, *N. tabacum* L., *Z. mays* L. and *O. sativa* L. Almost nothing or very little is known about *B. vulgaris* AQPs. Here we report the first investigation of AQPs in sugar beet. Twenty-eight *AQP* genes were identified, including seven PIPs, eight TIPs, nine NIPs, three SIPs and one XIP.

The number of AQPs genes in *B. vulgaris* is similar to that of *D. caryophyllus* (Morita et al., 2017), a closely related species belonging to the same family. *B. vulgaris* contains less AQP genes compared to plants that did undergo 1-3 times of whole genome duplication (WGD), fragment duplication (FD) and/or tandem duplication (TD), such as *G. max* L (Zhang et al., 2013), *Brassica rapa* (Tao et al., 2014), *Z. mays* L. (Chaumont et al., 2001), *O. sativa* L (Nguyen, Moon & Jung, 2013) and *P. trichocarpa* (Gupta & R, 2009). *B. Vulgaris* contains more AQP genes than basal plants such as *P. patens*, *S. moellendorffii* (Bowers et al., 2003; Doyle et al., 2008; Gupta & R, 2009; Yu & Yang, 2002; Zhang et al., 2013). Almost all subgroups of PIPs, TIPs, SIPs and XIPs are present in the Caryophyllaceae plants *B. vulgaris* and *D. caryophyllus* (Morita et al., 2017).

The relatively small number of *AQP* genes in sugar beet, compared to other higher plant species, is likely due to the fact that *B. vulgaris* did not undergo WGD. Moreover, the rapid expansion of NIP subfamily which led to the production of new subgroups, might have occurred after the divergence of the basal core dicot and core dicot. The divergence and proliferation of NIP subfamily may be an adaptive response to an ever-changing environment, playing crucial role in plants disease resistance and multi-stress (Liu & Zhu, 2010). Some studies also suggested that NIPs allow larger solutes, such as silicic acid, to permeate. *O. sativa* L genes *OsNIP2;1* (*Lsi1*), *OsNIP2;2* (*Lsi6*), and *Hordeum vulgare L. HvNIP2;1* (*HvLsi1*) transport silicon across the biomembrane and enhance the resistance of plants to biotic and abiotic stress (Chiba et al., 2009; Ma et al., 2006; Yamaji & Ma, 2009; Yamaji, Mitatni & Ma, 2008), and *O. sativa* L *OsNIP2;1* could also be permeable to water, urea, boric acid, arsenite (Ma et al., 2008; Mitani, Yamaji & Ma, 2008).

To analyze the evolutionary relationship and BvAQPs putative functions, an unrooted phylogenetic tree was constructed using aquaporins from *A. thaliana* (in which a complete set of AtAQP genes is well known) and *D. caryophyllus*, closely related species to *B. vulgaris* (Johanson & Kjellbom, 2001; Morita et al., 2017). During the process of cluster analysis, two carnation AQPs (DcSIP2;1 and DcNIP5;1) aroused our attention and made us confused in classification at once. Previously, the classification of DcSIP2;1 and DcNIP5;1 was not well resolved (Morita et al., 2017). In the case of DcSIP2;1, it shared the highest similarity of 67% with BvSIP1;1 and 51% with AtSIP1;1, and also shared the same NPA motifs, Ar/R selectivity filter and Froger’s position with BvSIP1;1. It clustered closer to the SIP1 subgroup. In the case of DcNIP5;1, it farther clustered to the NIP5 subgroup and clustered closer to the NIP6 subgroup. The closest homolog of DcNIP5;1 is BvNIP6;3 (similarity is 51%) and both proteins share the same Ar/R selectivity filter (S-I-A-R) with BvNIP6;3 and BvNIP6;1, with a similar Ar/R selectivity filter (A-I-A-R) to DcNIP6;1. Therefore, DcNIP5;1 belongs to NIP6 subgroup.

Alternative splicing is a mechanism by which genes can produce multiple transcript variants protein products, which allows in turn to increase the diversity of gene functions. Alternative splicing plays an important role in various processes such as development, response to pathogen and to various abiotic stresses (Bove et al., 2008; Gassmann, 2008; Jang et al., 2009; Reddy & Golovkin, 2010). A pair of splice variants (*BvSIP1;1 α* and *BvSIP1;1 β*) were found in BvAQPs. *BvSIP1;1 α* resulted from second exon skipping. First exon and second exon encoded the same ‘NPT’ type NPA motif, so *BvSIP1;1 α* and *BvSIP1;1 β* shared similar amino acid sequence and protein topology. These two genes had a similar expression pattern in different organs. It is possible that *BvSIP1;1 α* and *BvSIP1;1 β* may have redundant function. Exon skipping splicing were also found in *S. tuberosum*, while *St-SIP1;1 α* and *St-SIP1;1 β* also resulted from second exon skipping and shared similar expression pattern (Venkatesh, Yu & Park, 2013). However, the identified alternative splicing events found for BvAQPs genes were not reported in other plant species, suggesting that selective splicing may be species-specific.

NPA motifs, ar/R selectivity filter and Froger’s position were reported involved in substrate selection and transport activity. BvAQPs functions could be conferred based on the comparison of these residues with other plants AQPs. BvPIPs showed typical NPA motif, F-H-T-R highly conserved ar/R selectivity filter and Q-S-A-F-W Froger’s position, this composition is highly conserved in PIPs of *A. thaliana* (Johanson & Kjellbom, 2001), *Z. mays* (Chaumont et al., 2001), *S. lycopersicum* (Reuscher et al., 2013b), *P. trichocarpa* (Gupta & R, 2009), *G. max* (Zhang et al., 2013) and *B. rapa* (Tao et al., 2014). This composition of PIPs was reported to likely regulate root and leaf hydraulics, facilitate the CO_2_ diffusion, affect photosynthesis (Gupta & R, 2009). Therefore, *B. vulgaris* homologous PIPs may have similar roles in regulating water absorption, plant hydraulics and CO_2_ diffusion. Based on the SDPs analysis proposed by Hove and Bhave (Hove & Bhave, 2011) (Table 3; Fig. S2), all *B. vulgaris* PIPs represented urea-type SDPs, BvPIP1;1 and BvPIP1;2 represented boric acid-type SDPs, all PIP2s represented H_2_O_2_-type SDPs, thus supporting conserved functions. In addition, BvPIP1;2, BvPIP1;3 and BvPIP2;1 seemed to represent novel CO_2_-type SDPs (I/L/V-M-C-A-I/V-D/H/K-W-D-W) with the substitution of Ile for Met in SDP2 and the substitution of D to H/k in SDP6. Although the ar/R selectivity filter varied highly, plant TIPs were shown to transport water as efficiently as PIPs (Zhi et al., 2015). Additionally, BvTIP1;1, BvTIP1;2, BvTIP1;3, BvTIP3;1 and BvTIP4;1 contained dual conserved NPA motifs, H-I-A-V/R ar/R selectivity filter and T-S-A-Y-W Froger’s position, which had the same composition of *Citrus sinensis* AQPs, such as CsTIP1s, CsTIP3s showed to transport urea and H_2_O_2_ (Hove & Bhave, 2011). Compared to other TIPs, BvTIP2;1 and BvTIP2;2 had H-I-G-R ar/R selectivity filter, and this type ar/R selectivity filter was experimentally proven to transport formamide (Hove & Bhave, 2011), suggesting BvTIPs play a crucial role in transporting a wide range molecules. All TIPs (exception of BvTIP5;1) represented urea-type SDPs, indicting transporting urea function. BvTIP1;1 and BvTIP3;1 represented H_2_O_2_-type SDPs, indicting transporting H_2_O_2_ potential capacity.

Substrate selection specificity of NIPs is largely determined by two pores formed by NPA motifs and ar/R selectivity filter respectively. ‘W-V-A-R’ type ar/R selectivity filter were proven to be permeable to uncharged molecules i.e., glycerol, formamide and water, implying that BvNIP1;1 and BvNIP4;1 may have similar functions. ‘A/S/T-V/I-A/G-R’ type ar/R selectivity filter were reported can transport glycerol, formamide and larger solutes, like urea, boric, but were impermeable to water. Besides, NPS/NPV aqueous pore and A-I-G-R ar/R selectivity filter was known as a boric acid transporter in *A. thaliana* AtNIP5;1, and orthologs BvNIP5;1 can be involved in boron transport in *B. vulgaris*, suggesting that NIPs could be involved in the transport of larger solutes. Based on SDPs analysis, all NIPs (except BvNIP7;1) are potential urea transporters, while BvNIP4;2 is a potential H_2_O_2_ transporters, BvNIP5;2 and BvNIP6;1 are potential boric acid transporters and finaly BvNIP1;1 could represent a novel NH_3_-type SDPs with H replaced K/L/N/V at SDP2. It should be noticed that we were unable to find NIP2 subgroup and silicic acid-type SDP AQPs in sugar beet, suggesting that other types of SDPs AQPs could be involved in absorption of silicic during plant growth and development, in sugar beet. Sugar beet SIPs and XIPs exhibited great variety in ar/R selectivity filter and Froger’s position, with a variable first NPA motif compared to other AQPs subfamilies. It has been reported that *A. thaliana* SIPs (AtSIP1;1 and AtSIP1;2) localize to endoplasmic reticulum (ER) and facilitate water transport. SDPs analysis suggest that BvXIP1;1 can transport boric acid, it is thus possible that BvXIP has different role in *B. vulgaris*.

**Table 3:** Identified typical SDPs in BvAQPs.

Aquaporin		Specificity-determining positions								
		SDP1	SDP2	SDP3	SDP4	SDP5	SDP6	SDP7	SDP8	SDP9
**Typical NH_3_ transporter**		**F/T**	**K/L/N/V**	**F/T**	**V/L/T**	**A**	**D/S**	** A/H/L**	** E/P/S**	** A/R/T**
	BvNIP1;1	F	H	F	T	A	D	L	E	T
**Typical Boric Acid transporter**		**T/V**	**I/V**	**H/I**	**P**	**E**	**I/L**	** I/L/T**	**A/T**	** A/G/K/P**
	BvPIP1;2	T	I	H	P	E	L	L	T	P
	BvPIP1;3	T	I	H	P	E	L	L	T	P
	BvNIP5;2	T	I	H	P	E	L	L	A	P
	BvNIP6;1	T	I	H	P	E	L	L	A	P
	BvXIP1;1	T	I	H	P	E	L	L	T	P
**Typical CO2 transporter**		** I/L/V**	**I**	**C**	**A**	**I/V**	**D**	**W**	**D**	**W**
	BvPIP1;2	V	M	C	A	I	D	W	H	W
	BvPIP1;3	V	M	C	A	I	H	W	D	W
	BvPIP2;1	I	M	C	A	V	K	W	D	W
**Typical H2O2 transporters**		**A/S**	**A/G**	**L/V**	** A/F/L/T/V**	** I/L/V**	** H/I/L/Q**	**F/Y**	**A/V**	**P**
	BvPIP2;1	A	G	V	F	I	H	F	V	P
	BvPIP2;2	A	G	V	F	I	H	F	V	P
	BvPIP2;3	A	G	V	F	I	H	F	V	P
	BvPIP2;4	A	G	V	F	I	H	F	V	P
	BvTIP1;1	S	A	L	A	I	H	Y	V	P
	BvTIP3;1	A	A	L	T	I	H	Y	V	P
	BvNIP4;2	S	A	L	L	I	L	Y	V	P
**Typical silicic acid transporters**		**C/S**	**F/Y**	** A/E/L**	** H/R/Y**	**G**	** K/N/T**	**R**	** E/S/T**	** A/K/P/T**
	Not found									
**Typical urea transporters**		**H**	**P**	** F/I/L/T**	** A/C/F/L**	**L/M**	** A/G/P**	**G/S**	**G/S**	**N**
	BvPIP1;1	H	P	F	L	L	P	G	G	N
	BvPIP1;2	H	P	F	L	L	P	G	G	N
	BvPIP1;3	H	P	L	F	L	P	G	G	N
	BvPIP2;1	H	P	F	L	L	P	G	G	N
	BvPIP2;2	H	P	F	F	L	P	G	G	N
	BvPIP2;3	H	P	F	F	L	P	G	G	N
	BvPIP2;4	H	P	F	F	L	P	G	G	N
	BvTIP1;1	H	P	F	F	L	A	G	S	N
	BvTIP1;2	H	P	F	F	L	P	G	S	N
	BvTIP1;3	H	P	L	F	L	A	G	S	N
	BvTIP2;1	H	P	F	A	L	P	G	S	N
	BvTIP2;2	H	P	F	A	L	P	G	S	N
	BvTIP3;1	H	P	F	L	L	P	G	S	N
	BvTIP4;1	H	P	L	A	L	L	G	S	N
	BvNIP1;1	H	P	I	A	L	P	G	S	N
	BvNIP4;1	H	P	L	A	L	P	G	S	N
	BvNIP4;2	H	P	I	A	L	T	G	S	N
	BvNIP5;1	H	P	I	A	L	P	G	S	N
	BvNIP5;2	H	P	I	A	L	P	G	S	N
	BvNIP6;1	H	P	I	A	L	P	G	S	N
	BvNIP6;2	H	P	L	A	L	P	G	S	N
	BvNIP6;3	H	P	I	A	L	P	G	S	N

Sequence alignment revealed that *BvPIP1;1* codes for a truncated protein that lacks TM6 domain. The absence of TM6 domain possibly affects subcellular localization, proper folding, and oligomerization and transport activity of this truncated AQP in *B. vulgaris*. Similarly truncated AQP TdPIP2;1 has been reported to affect water transport activity in wheat (He et al., 2012; Hu, 2012). RNA-seq analysis revealed that *AQP* genes were expressed in all examined tissues of *B. vulgaris*, thus similar to previously reported data in maize (Chaumont et al., 2001), *A. Arabidopsis* (Quigley, 2001), tomato (Reuscher et al., 2013a) and potato (Venkatesh, Yu & Park, 2013). Total transcript abundance in the different examined tissues showed high expression levels in organs involved in water absorption, transport and evaporation, such as taproot and leaf, suggesting the main role of AQPs in water and nutrients transport. The RNA-seq data suggest that BvAQPs may play a key role in radial and axial water transportation in sugar beet, thus in agreement with Amodeo et al. (1999). Many AQPs show similar expression patterns, suggesting that they may act synergistically in some tissues. Co-expression of tandem PIP2-PIP1 dimers in Xenopus oocytes showed that they can form PIP2-PIP1 hetero-tetramers and synergistically increase water transport capacity (Bellati et al., 2010; Jozefkowicz et al., 2013; Jozefkowicz et al., 2015). *BvPIP1;3*, *BvTIP1;1* and *BvPIP2;4* were considerably abundant in taproot and are likely involved in water and nutrients transport in root. *BvPIP2;4*, *BvPIP1;3* and *BvTIP1;1* were abundant in inflorescence and seeding, and could be involved in monitoring the water balance in young tissues. According to their expression profiles, *BvTIP1;1*, *BvPIP1;3* and *BvTIP2;1* could play key roles in leaf hydraulics. *BvTIP3;1*, *BvPIP2;1*, *BvPIP1;3* and *BvTIP1;1* were considerably abundant in seed and therefore could be involved in water balance in seed. *BvPIP1;3* and *BvTIP1;1* show high expression levels in all examined tissues, while *BvTIP3;1* were only expressed in abundance in seed, and showed very low expression in other organs, suggesting a key role in seed development. *BvTIP3;1* closest homolog Arabidopsis *AtTIP3;1* and castor bean *RcTIP3;1* also shared similar expression pattern. *AtTIP3;1* was reported to be seed- and embryo-specific *AQP* (Johnson, Herman & Chrispeels, 1989). *RcTIP3;1* expressed preferentially in endosperm of developing seeds and considerably low in germinating seed (Zou et al., 2015). Moreover, *BvPIP2;2* and *BvTIP2;2* were highly expressed in seeding where they may control the water balance. In total, several *AQP* genes (like *BvTIP3;1*, *BvPIP2;1*, and *BvPIP1;3*) play a constitutive role and some *AQP* genes (like *BvTIP3;1*, *BvPIP2;2* and *BvTIP2;2*) play a specialized function in specific plants organs. In addition, it is noteworthy that several putative non-aqua transporter-encoding genes (i.e., *BvNIP7;1*, *BvNIP4;1*, *BvXIP1;1*, *BvNIP5;2*, *BvNIP6;1* and *BvSIP2;1*) were shown to be relatively high abundant in certain tissues. Compared to others tissues, transcript level of *BvNIP4;1*, *BvNIP5;2* and *BvNIP6;1* was considerably high in taproot. Similar to *BvNIP7;1*, *Arabidopsis AtNIP7;1* (specifically expressed in anthers) encode for a less efficient boric acid transporter, compared to *AtNIP5;1* and *AtNIP6;1*. It is possible that *BvNIP7;1* plays a role in B absorption and balance in inflorescence tissue. *BvXIP1;1* had relatively high expression level in leaf. Similarly, *BvXIP1;1* the closest homolog to *RcXIP1;1*, also showed high expression levels in leaf, but their exact functions remain unclear and further functional investigation are required.

In this study, a large number of *BvAQP* genes showed transcriptional changes when exposed to salt and heat stresses. For example, *BvTIP1;1* and *BvTIP2;1* showed strong induction (Log_2_-based value >1) after salt stress; *BvPIP2;2*, *BvTIP1;1*, *BvTIP2;1* and *BvXIP1;1* showed strong induction (Log_2_-based value >1) after heat stress, suggesting an extensive response of *BvAQP* genes to abiotic stress and a potential function for improving sugar beet resistance to abiotic stress. The results were consistent with previously reported data that showed that BvPIP2;2 expression was up-regulated in response to high salt stress (Skorupakłaput et al., 2015). Biochemical and genetic evidence has demonstrated that some *AQP* genes (like *TaAQP7*, *TaAQP8*, *TaNIP*, *MaPIP1;1*, *MusaPIP2;6* and *NtAQP1*) improve plants resistance to abiotic stress (Gao et al., 2010; Hu, 2012; Sade, 2010; Sreedharan, Shekhawat & Ganapathi, 2015; Xu et al., 2014; Zhou et al., 2012). Additionally, we noticed that *BvTIP1;1* and *BvTIP2;1* were strongly induced by both salt and heat stresses, suggesting that these two AQPs may play key roles in B. vulgaris adaption to environmental changes, e.g., heat and salt stresses.

## Conclusions

Twenty-eight *BvAQP* genes were identified based on genome data, chromosome distribution, phylogenetic, protein characteristics, gene structural, gene duplication, conserved motifs and RNA-seq expression analysis of *BvAQP* genes were further researched. The results suggested that AQPs had multiple functions in different tissues; several *BvAQP* genes responded to abiotic stresses and improved the plants’ resistance to abiotic stresses. The potential functions of BvAQPs were predicted and discussed based on NPA motifs, ar/R selectivity filter, Froger’s positions and SDP positions analysis. This study provide a useful resource for identifying and characterizing BvAQPs and a base for the breeding and genetic engineering of sugar beet.

##  Supplemental Information

10.7717/peerj.3747/supp-1 Fig. S1 Alignment of deduced amino acid sequences of BvAQPs

10.7717/peerj.3747/supp-2 Fig. S2 SDPs position of AQPs

10.7717/peerj.3747/supp-3 Table S1 Transcription abundance of BvAQP genes

## Additional Information and Declarations

### Data Availability

The following information was supplied regarding data availability: The raw data has been supplied as Supplementary Files.
