# Supplementary figures and images for: Genome-wide identification and characterization of aquaporin gene family in Beta vulgaris
Source: PeerJ. 2017 Sep 19;5:e3747. doi: 10.7717/peerj.3747 (PMC5609522; doi:10.7717/peerj.3747)

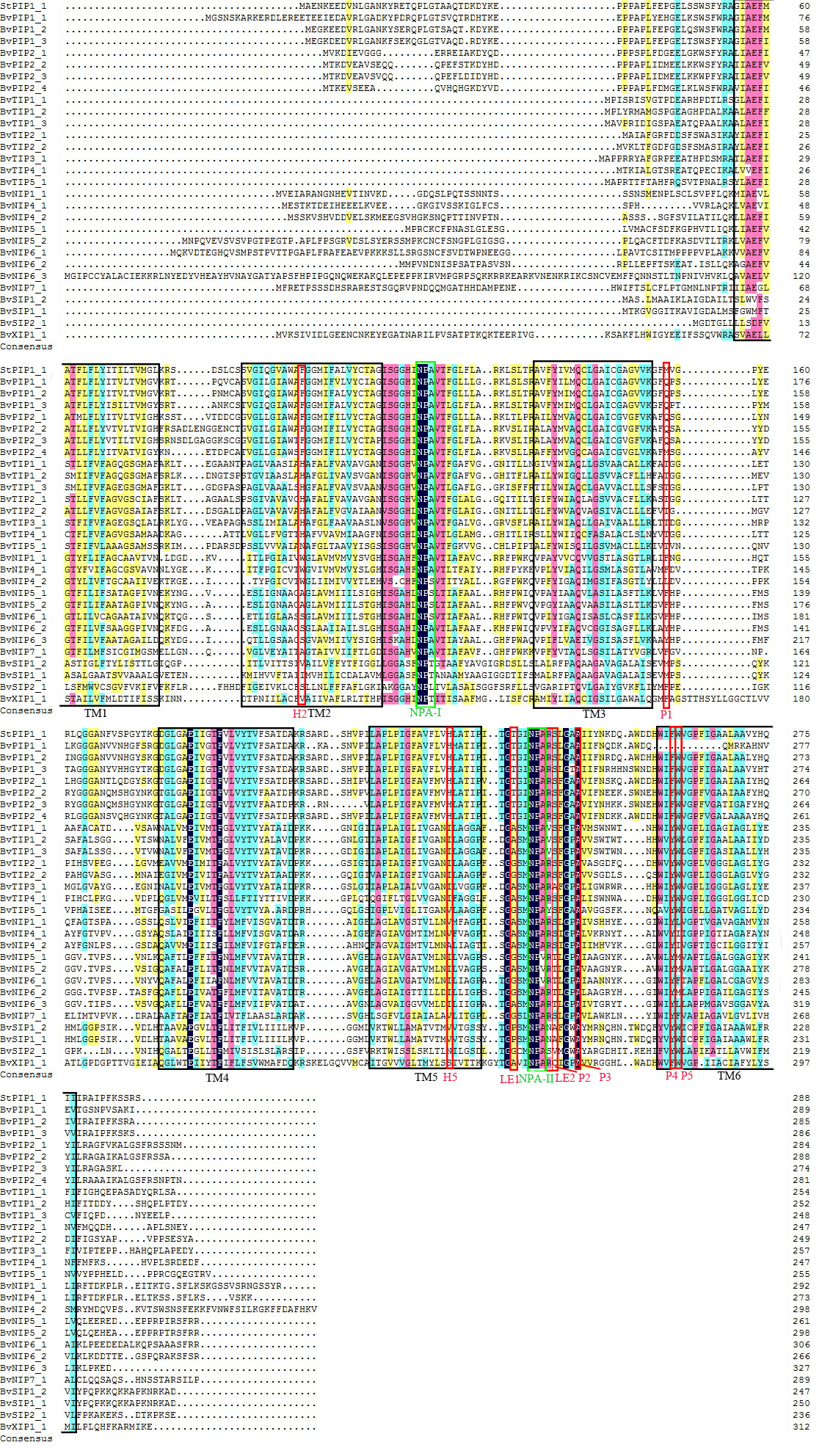


**Alignment of deduced amino acid sequences of BvAQPs**

Supplement: Fig. S1 [file peerj-05-3747-s001.docx]
