# Supplementary material for: Genome-wide identification and characterization of aquaporin gene family in Beta vulgaris
Source: PeerJ. 2017 Sep 19;5:e3747. doi: 10.7717/peerj.3747 (PMC5609522; doi:10.7717/peerj.3747)
Supplement: Fig. S2 [file peerj-05-3747-s002.pdf]

Ammonia (NH_3) Transporters(exact match wasn't found and three approximate match were found. approximate match means eight sites match on reference， and mismatch site highlighted in red)

```
BvNIP1;1     AFAVCRRFPWKQVPAYVVCQVVGSTLASGTLRLIFNGHQTQFAGTSPAGSSLQSLVIEFI
GmNOD26      AFASTRRFPLIQVPAYVVAQLLGSILASGTLRLLFMGNHDQFSGTVPNGTNLQAFVFEFI
TaTIP2;1     GLALGGQITILTGIFYWVAQLLGAIVGAFLVQFCT-GVATPTHGLS-GVGAFEGVVMEII
TaTIP2;2     GLALGGQITILTGIFYWVAQLLGAIVGAFLVQFCT-GVATPTHGLS-GVGAFEGVVMEII
AtTIP2;1     GLAVGGQITVITGVFYWIAQLLGSTAACFLLKYVTGGLAVPTHSVAAGLGSIEGVVMEII
AtTIP2;3     GLAIGGNITLITGFFYWIAQCLGSIVACLLLVFVTNGKSVPTHGVSAGLGAVEGVVMEIV

BvNIP1;1     ITFYLMFVISGVATDTR—AIGELAGLAVGSTVLLNVMFAGPISGASMNPARSLGPAIVS
GmNOD26      MTFFLMFVICGVATDNR--AVGEFAGIAIGSTLLLNVIIGGPVTGASMNPARSLGPAFVH
TaTIP2;1     VTFGLVYTVYATAADPKKGSLGTIAPIAIGFIVGANILVAGPFSGGSMNPARSFGPAVAS
TaTIP2;2     VTFGLVYTVYATAADPKKGSLGTIAPIAIGFIVGANILVAGPFSGGSMNPARSFGPAVAS
AtTIP2;1     ITFALVYTVYATAADPKKGSLGTIAPLAIGLIVGANILAAGPFSGGSMNPARSFGPAVAA
AtTIP2;3     VTFALVYTVYATAADPKKGSLGTIAPIAIGFIVGANILAAGPFSGGSMNPARSFGPAVVS

BvNIP1;1     HHYEGIWIYLVGPTVGAVAGAMVYNLIRFTDKPLREITKTGSFLKSKGSSVSRNGSSYR
GmNOD26      GEYEGIWIYLLAPVVGAIAGAWVYNIVRYTDKPLSETTKSASFLKGRAASK--------
TaTIP2;1     GDFTNIWVYWAGPLIGGGLAGVVYRYL-YM-CDDHSSVAGNDY----------------
TaTIP2;2     GDFTNIWIYWAGPLIGGGLAGVVYRYV-YM-CDDHSSVAGNDY----------------
AtTIP2;1     GDFSGHWVYWVGPLIGGGLAGLIYGNV-FMGSSEHVPLASADF----------------
AtTIP2;3     GDLSQIWIYWVGPLVGGALAGLIYGDV-FIGSYEAVETREIRV----------------
```

Boric Acid Transporters

```
BvPIP1;2     IQGIAWAFGGMIFALVYCTAGISGGHINPAVTFGLLLARKLSLTRAVFYMIMQCLGAICG
BvPIP1;3     IQGIAWAFGGMIFALVYCTAGISGGHINPAVTLGLFLARKLSLTRAVFYMIMQCLGAICG
BvNIP5;2     LIGNAACAGLAVMIIILSTGHISGAHLNPSLTIAFAALRHFPWVQVPGYIAAQVAASILA
BvNIP6;1     LIGLAASSGLAVMIIILSTGHISGAHLNPSVTIAFAALRHFPWTQVPIYIGAQISASLCA
BvXIP1;1     NIILACFVAIIVAFLRLTTHPISGGHINPTITISAAFMGLISFCRAMIYLIAQCIGSILG
NtXIP1;1α    NLIMSILIAIVITILLLAVVPVSGGHINPVISFSAALVGIISMSRAIIYMVAQCVGAILG
NtXIP1;1β    NLIMSILIAIVITILLLAVVPVSGGHINPVISFSAALVGIISMSRAIIYMVAQCVGAILG
StXIP1;1α    NLIMSILIAVVITILLLAVVPVSGGHINPVISFSAALVGIISMSRAIIYIVAQCLGAVLG
StXIP1;1β    NLIMSILIAVVITILLLAVVPVSGGHINPVISFSAALVGIISMSRAIIYIVAQCLGAVLG
ZmPIP1;1     IQGIAWSFGGMILALVYCTAGISG-HINPAVTFGLFLARKLSLTRAVFYIIMQCLGAICG
HvPIP1;3     IQGIAWSFGGMIFVLVYCTAGISGGHINPAVTFGLFLARKLSLTRAVFYIVMQCLGAICG
HvPIP1;4     IQGIAWSFGGMIFVLVYCTAGISGGHINPAVTFGLFLARKLSLTRAVFYIVMQCLGAICG
OsNIP2;1     QLGQSIAGGLIVTVMIYAVGHISGAHMNPAVTLAFAVFRHFPWIQVPFYWAAQFTGAICA
AtNIP5;1     LIGNAACAGLAVMIIILSTGHISGAHLNPSLTIAFAALRHFPWAHVPAYIAAQVSASICA
AtNIP6;1     LIGCAASAGLAVMIVILSTGHISGAHLNPAVTIAFAALKHFPWKHVPVYIGAQVMASVSA

BvPIP1;2     AGVVKG-FQPSLYEI-NGGGANV----------VNHGYSKGDGLGAEIVGTFVLVY-TVF
```

```
BvPIP1;3        AGVVKG-FQPTPYMT-AGGGANY----------VHHGYTKGDGLGAEIIGTFVLVY-TVF
BvNIP5;2        SLTLKGVFHPFMS-----GGVTV----------PSVSI--GQAFALEFLITFNLMFVVTA
BvNIP6;1        SFILKGVFHPIMS-----GGVTV----------PSVNY--VQAFALEFIIAFNLMFVVTA
BvXIP1;1        AWALQGMFAGSTTHSYLLGGCTLVVATLGPDGPTTVGIEIAQGLWTEIIYTFIFLF-SVW
NtXIP1;1α       ALALKAVVSSTIAQTFSLGGCTITVIAPGPNGPITVGLEMAQALWLEIFCTFVFLFASIW
NtXIP1;1β       ALALKAVVSSTIAQTFSLGGCTITVIAPGPNGPITVGLEMAQALWLEIFCTFVFLFASIW
StXIP1;1α       ALALRAVVSSSIEDTFSLGGCTVTIIAPGPNGPVTVGLETAQALWLEIFCTFVFLFASIW
StXIP1;1β       ALALRAVVSSSIEDTFSLGGCTVTIIAPGPNGPVTVGLETAQALWLEIFCTFVFLFASIW
ZmPIP1;1        RGVVKG-FQQGLYMG-NGGRRNV----------VAPGYTKGDGLGAEIVGTFILVY-TVF
HvPIP1;3        AGVVKG-FQTTLYQG-NGGGANS----------VAAGYTKGDGLGAEIVGTFVLVY-TVF
HvPIP1;4        AGVVKG-FQTTLYQG-NGGGANS----------VAAGYTKGDGLGAEIVGTFVLVY-TVF
OsNIP2;1        SFVLKAVIHPV-----DVIGTTT---------PVGPHW---HSLVVEVIVTFNMMFVTLA
AtNIP5;1        SFALKGVFHPFMS-----GGVTI----------PSVSL--GQAFALEFIITFILLFVVTA
AtNIP6;1        AFALKAVFEPTMS-----GGVTV----------PTVGL--SQAFALEFIISFNLMFVVTA
```

```
BvPIP1;2        SATDAKRSARDSHVPI--LAPLPIGFAVFLVHLATIPIT--GTGINPARSLGAAIIFNRD
BvPIP1;3        SATDAKRSARDSHVPI--LAPLPIGFAVFLVHLATIPIT--GTGINPARSLGTAIIFNRH
BvNIP5;2        VATDSRAV---GE-----LAGIAVGATVMLNILVAGPSS--GASMNPVRTLGPAVAAG--
BvNIP6;1        VATDTRAV---GE-----LAGIAVGATVMLNILIAGPAT--GGSMNPVRTLGPAIAAN--
BvXIP1;1        MAFDQKRSKELGQVVMCAITGVVVGLTMYLSSTVTTKKGYTGAVINPARCIGPAVVRGGH
NtXIP1;1α       MAYDHRQAKALGLVTVLSIVGIVLGLLVFISTTVTMKKGYAGAGMNPARCFGAAVVRGGH
NtXIP1;1β       MAYDHRQAKALGLVTVLSIVGIVLGLLVFISTTVTMKKGYAGAGMNPARCFGAAVVRGGH
StXIP1;1α       MAYDHRQAKALGHVTVLSIVGLVLGLLVFISTTVTAKKGYGGAGINPARCLGPAIIRGGH
StXIP1;1β       MAYDHRQAKALGHVTVLSIVGLVLGLLVFISTTVTAKKGYGGAGINPARCLGPAIIRGGH
ZmPIP1;1        SATDAKRRARDSHVPI--LAPLPIGFAVFLVHLATMGIT--GTGINPARSLGAAVIYNQH
HvPIP1;3        SATDAKRSARDSHVPI--LAPLPIGFAVFLVHLATIPIT--GTGINPARSLGAAIIYNKK
HvPIP1;4        SATDAKRSARDSHVPI--LAPLPIGFAVFLVHLATIPIT--GTGINPARSLGAAIIYNKK
OsNIP2;1        VATDTRAV---GE-----LAGLAVGSAVCITSIFAGAIS--GGSMNPARTLGPALASN--
AtNIP5;1        VATDTRAV---GE-----LAGIAVGATVMLNILVAGPST--GGSMNPVRTLGPAVASG--
AtNIP6;1        VATDTRAV---GE-----LAGIAVGATVMLNILIAGPAT--SASMNPVRTLGPAIAAN—
```

Carbon dioxide (CO_2) Transporters (exact match wasn't found and three approximate match were found. approximate match means eight sites match on reference， and mismatch site highlighted in red)

```
BvPIP1;2        GMIFALVYCTAGISGGHINPAVTFGLLLARKLSLTRAVFYMIMQCLGAICGAGVVKGFQP
BvPIP1;3        GMIFALVYCTAGISGGHINPAVTLGLFLARKLSLTRAVFYMIMQCLGAICGAGVVKGFQP
BvPIP2;1        GMIFILVYCTAGISGGHINPAVTFGLLLARKLTLPRAILYMVAQCLGAICGVGFVKAFQS
NtAQP1         GMIFALVYCTAGISGGHINPAVTFGLFLARKLSLTRAIFYIVMQCLGAICGAGVVKGFMV
AtPIP1;2        GMIFALVYCTAGISGGHINPAVTFGLFLARKLSLTRAVYYIVMQCLGAICGAGVVKGFQP
HvPIP2;1        GMIFVLVYCTAGVSGGHINPAVTFGLFLARKVSLIRALLYIIAQCLGAICGVGLVKGFQS
```

```
BvPIP1;2        SLYEINGGGANVVNHGYSKGDGLGAEIVGTFVLVYTVFSATDAKRSARDSHVPILAPLPI
BvPIP1;3        TPYMTAGGGANYVHHGYTKGDGLGAEIIGTFVLVYTVFSATDAKRSARDSHVPILAPLPI
```

```
BvPIP2;1      GLYNLHGGGANTLQDGYSKGTGLGAEIIGTFVLVYTVFSATDPKRSARDSHIPVLAPLPI
NtAQP1        GPYQRLGGGANVVNHGYTKGDGLGAEIIGTFVLVYTVFSATDAKRNARDSYVPILAPLPI
AtPIP1;2      KQYQALGGGANTIAHGYTKGSGLGAEIIGTFVLVYTVFSATDAKRNARDSHVPILAPLPI
HvPIP2;1      SYYVRYGGGANELSAGYSKGTGLAAEIIGTFVLVYTVFSATDPKRNARDSHIPVLAPLPI
```

```
BvPIP1;2      GFAVFLVHLATIPITGTGINPARSLGAAIIFNR-DQAWDHHWIFWVGPFIGAALAALYHQ
BvPIP1;3      GFAVFLVHLATIPITGTGINPARSLGTAIIFNRHHNSWNDHWIFWVGPFIGAALAAVYHT
BvPIP2;1      GFAVFMVHLATIPVTGTGINPARSFGAAVIFNS-KQAWDDHWIFWVGPFIGAAIAAIYHQ
NtAQP1        GFAVFLVHLATIPITGTGINPARSLGAAIIYNT-DQAWDDHWIFWVGPFIGAALAAVYHQ
AtPIP1;2      GFAVFLVHLATIPITGTGINPARSLGAAIIFNK-DNAWDDHWVFWVGPFIGAALAALYHV
HvPIP2;1      GFAVFMVHLATIPITGTGINPARSLGAAVIYNT-DKAWDDQWIFWVGPLIGAAIAAAYHQ
```

H_2O_2 Transporters

```
BvPIP2;1      LVYCTAGISGGHINPAVTFGLLLARKLTLPRAILYMVAQCLGAICGVGFVKAFQ------
BvPIP2;2      LVYCTAGISGGHINPAVTFGLFLARKVSLIRALAYMVAQCLGAICGVGFVKAFQ------
BvPIP2;3      LVYCTAPISGGHINPAVTFGLFLARKVSLIRALAYMVAQCLGAICGVGFVKAFQ------
BvPIP2;4      LVYCTAGISGGHINPAVTFGLFLARKVSLLRAFFYMVGQCAGAICGVGLVKAFM------
BvTIP1;1      AVAVGANISGGHVNPAVTFGAFVGGNITLLNGIVYWIAQLLGSVAACALLKFAT------
BvTIP3;1      AVAASLNVSGGHVNPAVTFGALVGGRVSFLRAILYWIAQLLGAIVAALLLRLTT------
BvNIP4;2      VVYTLEHVSC-HFNPSVTITYALLRGFPWKQVPFYIGAQIMGSIFASGTLYLLL------
NtXIP1;1α    LLLAVVPVSGGHINPVISFSAALVGIISMSRAIIYMVAQCVGAILGALALKAVVSSTIAQ
NtXIP1;1β    LLLAVVPVSGGHINPVISFSAALVGIISMSRAIIYMVAQCVGAILGALALKAVVSSTIAQ
SlXIP1;1α    LLLAVVPVSGGHINPVISFSAALVGIISMSRAIIYIVAQCVGAILGALALRAVVSSSIED
SlXIP1;1β    LLLAVVPVSGGHINPVISFSAALVGIISMSRAIIYIVAQCVGAILGALALRAVVSSSIED
StXIP1;1α    LLLAVVPVSGGHINPVISFSAALVGIISMSRAIIYIVAQCLGAVLGALALRAVVSSSIED
StXIP1;1β    LLLAVVPVSGGHINPVISFSAALVGIISMSRAIIYIVAQCLGAVLGALALRAVVSSSIED
AtNIP1;2      LVYSLGHISGAHFNPAVTIAFASCGRFPLKQVPAYVISQVIGSTLAAATLRLLF------
AtPIP2;4      LVYCTAGISGGHINPAVTVGLFLARKVSLVRTVLYIVAQCLGAICGCGFVKAFQ------
AtPIP2;1      LVYCTAGISGGHINPAVTFGLFLARKVSLPRALLYIIAQCLGAICGVGFVKAFQ------
AtTIP2;3      GVSIAANISGGHLNPAVTLGLAIGGNITLITGFFYWIAQCLGSIVACLLLVFVT------
TgTIP1;1      AVSVGANISGGHVNPAVTFGAFLGGNITLLRGVLYIIAQLLGSSVACLLLRFTT------
TgTIP1;2      AVSVGANISGGHVNPAVTFGASLGGNITLLRGVLYIIAQLLGSSVACLLLRFTT------
AtTIP1;1      AVSVGANISGGHVNPAVTFGAFIGGNITLLRGILYWIAQLLGSVVACLILKFAT------
AtTIP1;2      AVSVGANISGGHVNPAVTFGVLLGGNITLLRGILYWIAQLLGSVAACFLLSFAT------
```

```
BvPIP2;1      -----SGLYNLHGGGAN---T-LQDGYSKGTGLGAEIIGTFVLVYTVFS-ATDPKRSARD
BvPIP2;2      -----SAYYDRYGGGAN---Q-MSHGYNKGTGLGAEIIGTFVLVYTVFA-ATDPKRSARD
BvPIP2;3      -----SAYYDRYGGGAN---Q-MSHGYNKGTGLGAEIIGTFVLVYTVFA-ATDPKR----
BvPIP2;4      -----SGAYVRLGGGAN---S-VQHGYNKGTALGAEIIGTFVLVYTVFS-ATDPKRSARD
BvTIP1;1      -----GG----LETAAF---A-CATDVSAWNALVMEIVMTFGLVYTVYATAIDPKK----
BvTIP3;1      -----DG----MRPMGL---G-VAYGEGNINALVLEIVMTFGLVYTVYATAIDPKR----
BvNIP4;2      ---------DVPPKAYF---GNLPSG-SDAQAVVMEIIISFILMFVIFGTAFDERA----
```

```
NtXIP1;1α     TFSLGGCTITVIAPGPN---GPITVGLEMAQALWLEIFCTFVFLFASIWMAYDHRQAKAL
NtXIP1;1β     TFSLGGCTITVIAPGPN---GPITVGLEMAQALWLEIFCTFVFLFASIWMAYDHRQAKAL
SlXIP1;1α     TFSLGGCTVTIIAPGPN---GPVIVGLETAQALWLEIFCTFVFLFASIWMAYDHRQAKAL
SlXIP1;1β     TFSLGGCTVTIIAPGPN---GPVIVGLETAQALWLEIFCTFVFLFASIWMAYDHRQAKAL
StXIP1;1α     TFSLGGCTVTIIAPGPN---GPVTVGLETAQALWLEIFCTFVFLFASIWMAYDHRQAKAL
StXIP1;1β     TFSLGGCTVTIIAPGPN---GPVTVGLETAQALWLEIFCTFVFLFASIWMAYDHRQAKAL
AtNIP1;2       -----GLDQDVCSGKHDVFVGTLPSG-SNLQSFVIEFIITFYLMFVISGVATDNRA----
AtPIP2;4       -----SSYYTRYGGGAN---E-LADGYNKGTGLGAEIIGTFVLVYTVFS-ATDPKRNARD
AtPIP2;1       -----SSYYTRYGGGAN---S-LADGYSTGTGLAAEIIGTFVLVYTVFS-ATDPKRSARD
AtTIP2;3       -----NG----KSVPTH---G-VSAGLGAVEGVVMEIVVTFALVYTVYATAADPKK----
TgTIP1;1       -----G-----LGTGTF---G-LVAGVSVWSGLVMEIVMTFGLVYTVYATAVDPKK----
TgTIP1;2       -----G-----LGTGTF---G-LVAGVSVWSGLVMEIVMTFGLVYTVYATAVDPKK----
AtTIP1;1       -----GG----LAVPAF---G-LSAGVGVLNAFVFEIVMTFGLVYTVYATAIDPKN----
AtTIP1;2       -----GG----EPIPAF---G-LSAGVGSLNALVFEIVMTFGLVYTVYATAVDPKN----

BvPIP2;1       SHIPVLAPLPIGFAVFMVHLATIPVT------GTGINPARSFGAAVIFNSKQAWDDHWIF
BvPIP2;2       SHVPVLAPLPIGFAVFMVHLATIPIT------GTGINPARSFGAAVIFNEEKSWNEHWIF
BvPIP2;3       --RNVLAPLPIGFAVFMVHLATIPIT------GTGINPARSFGAAVIYNHKKSWNEHWIF
BvPIP2;4       SHVPILAPLPIGFAVFMVHLATIPIT------GTGINPARSFGAAVIFNDKKAWDDHWIF
BvTIP1;1       GNIGIIAPLAIGLIVGANILAGGAFD------GASMNPAVSFGPAVM---SWNWTNHWIY
BvTIP3;1       GSLGTIAPLAIALVVGANILVGGPFD------GASMNPARAFGPALI---GWRWRHHWIY
BvNIP4;2       --HNQFAGVAIGMTVLMNALIAGTIS------GASMNPARSIGPAII---MHVYKGLWIY
NtXIP1;1α     GLVTVLS--IVGIVLGLLVFISTTVTMKKGYAGAGMNPARCFGAAVV-RGGHLWDGHWIF
NtXIP1;1β     GLVTVLS--IVGIVLGLLVFISTTVTMKKGYAGAGMNPARCFGAAVV-RGGHLWDGHWIF
SlXIP1;1α     GHVTVLS--IVGLVLGLLVFISTTVTAKKGYGGAGINPARCLGPAII-RGGHLWDGHWIF
SlXIP1;1β     GHVTVLS--IVGLVLGLLVFISTTVTAKKGYGGAGINPARCLGPAII-RGGHLWDGHWIF
StXIP1;1α     GHVTVLS--IVGLVLGLLVFISTTVTAKKGYGGAGINPARCLGPAII-RGGHLWDGHWIF
StXIP1;1β     GHVTVLS--IVGLVLGLLVFISTTVTAKKGYGGAGINPARCLGPAII-RGGHLWDGHWIF
AtNIP1;2       --IGELAGLAVGSTVLLNVIIAGPVS------GASMNPGRSLGPAMV---YSCYRGLWIY
AtPIP2;4       SHVPVLAPLPIGFAVFMVHLATIPIT------GTGINPARSFGAAVIYNNEKAWDDQWIF
AtPIP2;1       SHVPVLAPLPIGFAVFMVHLATIPIT------GTGINPARSFGAAVIYNKSKPWDDHWIF
AtTIP2;3       GSLGTIAPIAIGFIVGANILAAGPFS------GGSMNPARSFGPAVV---SGDLSQIWIY
TgTIP1;1       GDIGTIAPIAIGFIVGANILVGGAFT------GASMNPAIAFGPALV---SWSWTSHWVY
TgTIP1;2       GDIGTIAPIAIGFIVGANILVGGAFT------GASMNPAIAFGPALV---SWSWASHWVY
AtTIP1;1       GSLGTIAPIAIGFIVGANILAGGAFS------GASMNPAVAFGPAVV---SWTWTNHWVY
AtTIP1;2       GSLGTIAPIAIGFIVGANILAGGAFS------GASMNPAVAFGPAVV---SWTWTNHWVY

BvPIP2;1       WVGPFIGAAIAAIYHQYI-----LRA---GFVKALGSFRSSSNM----------------
BvPIP2;2       WVGPFVGAAIAAFYHQYI-----LRA---GAIKALGSFRSSA------------------
BvPIP2;3       WVGPFVGATIGAFYHQYI-----LRA----GASKL-------------------------
BvPIP2;4       WVGPFVGALAAAAYHQYI-----LRA---AAIKALGSFRSNPTN----------------
BvTIP1;1       WVGPLIGAGIAGLIYEFI-----FIG------HQEPASADYQRLSA--------------
```

BvTIP3;1	WVGPLIGGGLAGLIYEFI-----VIPTEPPHAHQPLAPEDY-------------------
BvNIP4;2	IVGPTIGCILGGITYISMRYMDQVPS--KVTSWSNSFEKKFVNWFSILKGKFFDAFHKVH
NtXIP1;1α	WVGPTIAC-VAFYVYTKI-----IPP---KHFHADGYKYDFIGVVKASFGLHE-------
NtXIP1;1β	WVGPTIAC-VAFYVYTKI-----IPP---KHFHADGYKYDFIGVVKASFGLHE-------
SlXIP1;1α	WVGPTIGC-VAFYVYTKI-----IPT--KHFLAEYGFKHDFVGVVKALSNV---------
SlXIP1;1β	WVGPTIGC-VAFYVYTKI-----IPT--KHFLAEYGFKHDFVGVVKALSNV---------
StXIP1;1α	WVGPTIGC-VAFYVYTKI-----IPP--KHFLAEYGFKHDFVGVVKALSNV---------
StXIP1;1β	WVGPTIGC-VAFYVYTKI-----IPP--KHFLAEYGFKHDFVGVVKALSNV---------
AtNIP1;2	IVSPIVGAVSGAWVYNMV-----RYT--DKPLREITKSGSFLKTVRNGSSR---------
AtPIP2;4	WVGPMIGAAAAAFYHQFI-----LRA---AAIKALGSFGSFGSFRSFA------------
AtPIP2;1	WVGPFIGAAIAAFYHQFV-----LRA---SGSKSLGSFRSAANV----------------
AtTIP2;3	WVGPLVGGALAGLIYGDV-----FIG-----SYEAVETREIRV-----------------
TgTIP1;1	WVGPLIGGGIAGIVYDLF-----FIN----STHEQLPSTDY-------------------
TgTIP1;2	WVGPLIGGGIAAIVYDLF-----FIS----GTHEQLPTTDY-------------------
AtTIP1;1	WAGPLVGGGIAGLIYEVF-----FIN----TTHEQLPTTDY-------------------
AtTIP1;2	WAGPLIGGGLAGIIYDFV-----FID---ENAHEQLPTTDY-------------------

Silicic Acid Transporters

Not found.

Urea Transporters

BvPIP1;1	VLVYCIAGISGGHINPAVTFGLLLGRKLSLSRAVFYILMQCLGAICGAGVVKGFQPSLYE
BvPIP1;2	ALVYCTAGISGGHINPAVTFGLLLARKLSLTRAVFYMIMQCLGAICGAGVVKGFQPSLYE
BvPIP1;3	ALVYCTAGISGGHINPAVTLGLFLARKLSLTRAVFYMIMQCLGAICGAGVVKGFQPTPYM
BvPIP2;1	ILVYCTAGISGGHINPAVTFGLLLARKLTLPRAILYMVAQCLGAICGVGFVKAFQSGLYN
BvPIP2;2	ILVYCTAGISGGHINPAVTFGLFLARKVSLIRALAYMVAQCLGAICGVGFVKAFQSAYYD
BvPIP2;3	ILVYCTAPISGGHINPAVTFGLFLARKVSLIRALAYMVAQCLGAICGVGFVKAFQSAYYD
BvPIP2;4	ILVYCTAGISGGHINPAVTFGLFLARKVSLLRAFFYMVGQCAGAICGVGLVKAFMSGAYV
BvTIP1;1	VAVAVGANISGGHVNPAVTFGAFVGGNITLLNGIVYWIAQLLGSVAACALLKFATGGL--
BvTIP1;2	VAVSVGANISGGHVNPAVTFGAFVGGHITFLRAILYWIGQLLGSVVACFLLHFATGGM--
BvTIP1;3	VAVSVAANVSGGHVNPAVTLGAFLGGKISFFRTILYWIAQCLGAVVACLLLSFSTGGL--
BvTIP2;1	VAVAVGANISGGHVNPAVTFGLALGGQITILTGIFYWIAQLAGSVVACFLLKASTGGL--
BvTIP2;2	VGVAIAANVSGGHLNPAVTFGLAIGGNITLLTGLFYWVAQVAGSIVACLLLEFVT-GM--
BvTIP3;1	AAVAASLNVSGGHVNPAVTFGALVGGRVSFLRAILYWIAQLLGAIVAALLLRLTTDGM--
BvTIP4;1	VMIAAGFNISGGHLNPAVTLGLAMGGHITLIRSLLYWIIQCFASALACLSLNYVTGGL--
BvNIP1;1	VMVYSVGHISGAHFNPAVTIAFAVCRRFPWKQVPAYVVCQVVGSTLASGTLRLIFNGH--
BvNIP4;1	VMVYSLGHISGAHFNPAVTLTFAIYRHFPYKEVPLYVIAQILGSMLASGTLAVMFDVT--
BvNIP4;2	IVVYTLEHVS-CHFNPSVTITYALLRGFPWKQVPFYIGAQIMGSIFASGTLYLLLDVP--
BvNIP5;1	IIILSIGHISGAHLNPSLTIAFAALRHFPWIQVPAYIAAQVLASILASFTLKLVFHPF--
BvNIP5;2	IIILSTGHISGAHLNPSLTIAFAALRHFPWVQVPGYIAAQVAASILASLTLKGVFHPF--
BvNIP6;1	IIILSTGHISGAHLNPSVTIAFAALRHFPWTQVPIYIGAQISASLCASFILKGVFHPI--
BvNIP6;2	IAILSLGHISGAHINPAVTLAFAAFKHFPWIQVPVYIFAQVCGGISAGFLLKLAYHPFM-

```
BvNIP6;3      IIVYSIGHISKAHLNPAVTIAYAALGHFPWAQVPIFLVAEIVGSISASFLVKAAYHPF--
NtXIP1;1α     ILLLAVVPVSGGHINPVISFSAALVGIISMSRAIIYMVAQCVGAILGALALKAVVSSTIA
NtXIP1;1β     ILLLAVVPVSGGHINPVISFSAALVGIISMSRAIIYMVAQCVGAILGALALKAVVSSTIA
StXIP1;1α     ILLLAVVPVSGGHINPVISFSAALVGIISMSRAIIYIVAQCLGAVLGALALRAVVSSSIE
StXIP1;1β     ILLLAVVPVSGGHINPVISFSAALVGIISMSRAIIYIVAQCLGAVLGALALRAVVSSSIE
CpNIP1        VMIYAVGHISGAHMNPAVTTAFAATRHFPWKQVPLYAAAQLSGATCAAFTLRLLLHPI--
OsNIP2;1      VMIYAVGHISGAHMNPAVTLAFAVFRHFPWIQVPFYWAAQFTGAICASFVLKAVIHPV--
AtNIP6;1      IVILSTGHISGAHLNPAVTIAFAALKHFPWKHVPVYIGAQVMASVSAAFALKAVFEPTM-
NtAQP1        ALVYCTAGISGGHINPAVTFGLFLARKLSLTRAIFYIVMQCLGAICGAGVVKGFMVGPYQ
ZmPIP1;5      ALVYCTAGISGGHINPAVTFGLFLARKLSLTRALFYMVMQCLGAICGAGVVKGFQEGLYM
AtTIP5;1      SSVYISWNVSGGHVNPAVTFAMAVAGRISVPTAMFYWTSQMIASVMACLVLKVTVMEQ--
AtTIP4;1      VMI-SAGHISGGHLNPAVTLGLLLGGHISVFRAFLYWIDQLLASSAACFLLSYLTGGM--
NtTIPa        VTISAGFRISGGHLNPAVTLGLCMGGHITVFRSILYWIDQLLASVAACALLNYLTAGL--
AtTIP1;1      VAVSVGANISGGHVNPAVTFGAFIGGNITLLRGILYWIAQLLGSVVACLILKFATGGL--
AtTIP1;2      VAVSVGANISGGHVNPAVTFGVLLGGNITLLRGILYWIAQLLGSVAACFLLSFATGGE--
AtTIP1;3      VAVSVGANVSGGHVNPAVTFGAFIGGNITLLRAILYWIAQLLGAVVACLLLKVSTGGM--
AtTIP2;1      VAVAIGANISGGHVNPAVTFGLAVGGQITVITGVFYWIAQLLGSTAACFLLKYVTGGL--

BvPIP1;1      ------------LKGGGANVVNHGFSRGDGLGAEIVGTFVLVYTVF-SATDAKRKA--SN
BvPIP1;2      ------------INGGGANVVNHGYSKGDGLGAEIVGTFVLVYTVF-SATDAKRSARDSH
BvPIP1;3      ------------TAGGGANYVHHGYTKGDGLGAEIIGTFVLVYTVF-SATDAKRSARDSH
BvPIP2;1      ------------LHGGGANTLQDGYSKGTGLGAEIIGTFVLVYTVF-SATDPKRSARDSH
BvPIP2;2      ------------RYGGGANQMSHGYNKGTGLGAEIIGTFVLVYTVF-AATDPKRSARDSH
BvPIP2;3      ------------RYGGGANQMSHGYNKGTGLGAEIIGTFVLVYTVF-AATDPKR------
BvPIP2;4      ------------RLGGGANSVQHGYNKGTALGAEIIGTFVLVYTVF-SATDPKRSARDSH
BvTIP1;1      ---------------ETAAFACATDVSAWNALVMEIVMTFGLVYTVYATAIDPKK----GN
BvTIP1;2      ---------------EVSAFALSGGVTSWNALVFEIVMTFGLVYTVYALAVDPKK----GN
BvTIP1;3      ---------------PTSAFALSSGVTVWNALVFEIVMTFGLVYTVYATAVDPKR----GD
BvTIP2;1      ---------------TTPIHSVPEGLGVMEAVVMEIMITFALVYTVYATAVDPKK----GS
BvTIP2;2      ---------------GVPAHGVASGMNAIEGIVMEIVITFALVYTVYATAADPKK----GQ
BvTIP3;1      ---------------RPMGLGVAYGEGNINALVLEIVMTFGLVYTVYATAIDPKR----GS
BvTIP4;1      ---------------TTPIHCLPKGVDPLQGLVMEVILTFSLLFTIYTTIVDPKK----GP
BvNIP1;1      ---------------QTQFAGTSPAGSSLQSLVIEFIITFYLMFVISGVATDTRA------
BvNIP4;1      ---------------PKAYFGTVPVGSYAQSLAIEIIISFLLMFVISGVATDARA------
BvNIP4;2      ---------------PKAYFGNLPSGSDAQAVVMEIIISFILMFVIFGTAFDERA------
BvNIP5;1      ----------------MSGGVTVPSVNLKQAFTLEFFITFNLMFVVTAVATDTRA------
BvNIP5;2      ----------------MSGGVTVPSVSIGQAFALEFLITFNLMFVVTAVATDSRA------
BvNIP6;1      ----------------MSGGVTVPSVNYVQAFALEFIIAFNLMFVVTAVATDTRA------
BvNIP6;2      ---------------SGGGTVPSPTASFGQAFLLEIVATFFLMFVITAVATDTRA------
BvNIP6;3      ----------------MFGGVTIPSVSVGQAFLLEFVATFFLMFVIIPVATDATA------
NtXIP1;1α     QTFSLGGCTITVIAPGPNGPITVGLEMAQALWLEIFCTFVFLFASIWMAYDHRQAKALGL
NtXIP1;1β     QTFSLGGCTITVIAPGPNGPITVGLEMAQALWLEIFCTFVFLFASIWMAYDHRQAKALGL
```

```
StXIP1;1α     DTFSLGGCTVTIIAPGPNGPVTVGLETAQALWLEIFCTFVFLFASIWMAYDHRQAKALGH
StXIP1;1β     DTFSLGGCTVTIIAPGPNGPVTVGLETAQALWLEIFCTFVFLFASIWMAYDHRQAKALGH
CpNIP1        ---------------KHLGTTTPSGSDLQALVMEIVVTFSMMFVTCAVATDTKA------
OsNIP2;1      ---------------DVIGTTTPVGPHWHSLVVEVIVTFNMMFVTLAVATDTRA------
AtNIP6;1      --------------SGGVTVPTVGLS--QAFALEFIISFNLMFVVTAVATDTRA------
NtAQP1        ------------RLGGGANVVNHGYTKGDGLGAEIIGTFVLVYTVF-SATDAKRNARDSY
ZmPIP1;5      ------------GAGGGANAVNPGYTKGDGLGAEIVGTFVLVYTVF-SATDAKRSARDSH
AtTIP5;1      --------------HVPIYKIAGEMTGFGASVLEGVLAFVLVYTVF-TASDPRR----GL
AtTIP4;1      --------------GTPVHTLASGVSYTQGIIWEIILTFSLLFTVYATIVDPKK----GS
NtTIPa        --------------ETPVHTLANGVSYGQGIIMEVILTFSLLFTVYTTIVDPKK----GI
AtTIP1;1      --------------AVPAFGLSAGVGVLNAFVFEIVMTFGLVYTVYATAIDPKN----GS
AtTIP1;2      --------------PIPAFGLSAGVGSLNALVFEIVMTFGLVYTVYATAVDPKN----GS
AtTIP1;3      --------------ETAAFSLSYGVTPWNAVVFEIVMTFGLVYTVYATAVDPKK----GD
AtTIP2;1      --------------AVPTHSVAAGLGSIEGVVMEIIITFALVYTVYATAADPKK----GS

BvPIP1;1      VPILA--PLPIGFAVFLVHMATIP--ITGTGINPARSLGAAIIFNQDK-AWDQQ------
BvPIP1;2      VPILA--PLPIGFAVFLVHLATIP--ITGTGINPARSLGAAIIFNRDQ-AWDHHWIFWVG
BvPIP1;3      VPILA--PLPIGFAVFLVHLATIP--ITGTGINPARSLGTAIIFNRHHNSWNDHWIFWVG
BvPIP2;1      IPVLA--PLPIGFAVFMVHLATIP--VTGTGINPARSFGAAVIFNSKQ-AWDDHWIFWVG
BvPIP2;2      VPVLA--PLPIGFAVFMVHLATIP--ITGTGINPARSFGAAVIFNEEK-SWNEHWIFWVG
BvPIP2;3      RNVLA--PLPIGFAVFMVHLATIP--ITGTGINPARSFGAAVIYNHKK-SWNEHWIFWVG
BvPIP2;4      VPILA--PLPIGFAVFMVHLATIP--ITGTGINPARSFGAAVIFNDKK-AWDDHWIFWVG
BvTIP1;1      IGIIA--PLAIGLIVGANILAGGA--FDGASMNPAVSFGPAVMSW----NWTNHWIYWVG
BvTIP1;2      LGTIA--PIAIGFIVGANILAGGP--FDGASMNPAVSFGPAVVSW----TWTHHWVYWLG
BvTIP1;3      IGIIA--PLAIGLIVGANILAGGA--FDGASMNPAVSFGPAVVSW----TWNNHWVYWLG
BvTIP2;1      IGTIA--PIAIGFIVGANILAAGP--FSGGSMNPARSFGPAVASG----DFQDHWVYWVG
BvTIP2;2      IGIVA--PIAIGFIVGANILAAGP--FSGGSMNPARSFGPAVVSG----DLSQSWIYWVG
BvTIP3;1      LGTIA--PLAIALVVGANILVGGP--FDGASMNPARAFGPALIGW----RWRHHWIYWVG
BvTIP4;1      LQTQG--IFLTGLVVGANIFAGGL--FSGASMNPARSFGPALISW----NWADHWIYWVG
BvNIP1;1      IGELA--GLAVGSTVLLNVMFAGP--ISGASMNPARSLGPAIVSH----HYEGIWIYLVG
BvNIP4;1      IGEFA--GIAVGMTIMLNVFVAGP--ISGASMNPARSIGPALVKR----NYTALWVYIIG
BvNIP4;2      HNQFA--GVAIGMTVLMNALIAGT--ISGASMNPARSIGPAIIMH----VYKGLWIYIVG
BvNIP5;1      VGELA--GIAVGATVMLNILVAGP--SSGGSMNPVRTLGPAIAAG----NYKAVWLYMVA
BvNIP5;2      VGELA--GIAVGATVMLNILVAGP--SSGASMNPVRTLGPAVAAG----NYRAVWIYMVA
BvNIP6;1      VGELA--GIAVGATVMLNILIAGP--ATGGSMNPVRTLGPAIAAN----NYKGIWIYFTA
BvNIP6;2      VGELA--GIAIGTTILLDILLIGP--STGGSMNPARTLGPALAAG----RYHGIWIYMLA
BvNIP6;3      VGNLA--GVAIGGVVMLDILIIGP--ATGASMNPARTLGPAIVTG----RYTGIWIYLLA
NtXIP1;1α     VTVLSIVGIVLGLLVFISTTVTMKKGYAGAGMNPARCFGAAVVRGGH--LWDGHWIFWVG
NtXIP1;1β     VTVLSIVGIVLGLLVFISTTVTMKKGYAGAGMNPARCFGAAVVRGGH--LWDGHWIFWVG
StXIP1;1α     VTVLSIVGLVLGLLVFISTTVTAKKGYGGAGINPARCLGPAIIRGGH--LWDGHWIFWVG
StXIP1;1β     VTVLSIVGLVLGLLVFISTTVTAKKGYGGAGINPARCLGPAIIRGGH--LWDGHWIFWVG
CpNIP1        VGELA--GLAVGSAVCITSILAGP--VSGGSMNPVRTLGPAMASD----NYKGLWVYFVG
```

OsNIP2;1	VGELA--GLAVGSAVCITSIFAGA--ISGGSMNPARTLGPALASN----KFDGLWIYFLG
AtNIP6;1	VGELA--GIAVGATVMLNILIAGP--ATSASMNPVRTLGPAIAAN----NYRAIWVYLTA
NtAQP1	VPILA--PLPIGFAVFLVHLATIP--ITGTGINPARSLGAAIIYNTDQ-AWDDHWIFWVG
ZmPIP1;5	VPILA--PLPIGFAVFLVHLATIP--ITGTGINPARSLGAAIVYNRSH-AWNDHWIFWVG
AtTIP5;1	PLAVG--PIFIGFVAGANVLAAGP--FSGGSMNPACAFGSAMVYG----SFKNQAVYWVG
AtTIP4;1	LDGFG--PLLTGFVVGANILAGGA--FSGASMNPARSFGPALVSG----NWTDHWVYWVG
NtTIPa	LEGMG--PLLTGLVVGANIMAGGP--FSGASMNPARSFGPAFVSG----IWTDHWVYWVG
AtTIP1;1	LGTIA--PIAIGFIVGANILAGGA--FSGASMNPAVAFGPAVVSW----TWTNHWVYWAG
AtTIP1;2	LGTIA--PIAIGFIVGANILAGGA--FSGASMNPAVAFGPAVVSW----TWTNHWVYWAG
AtTIP1;3	IGIIA--PLAIGLIVGANILVGGA--FDGASMNPAVSFGPAVVSW----IWTNHWVYWVG
AtTIP2;1	LGTIA--PLAIGLIVGANILAAGP--FSGGSMNPARSFGPAVAAG----DFSGHWVYWVG
