# Supplementary material for: Genome-wide identification and characterization of aquaporin gene family in Beta vulgaris
Source: PeerJ. 2017 Sep 19;5:e3747. doi: 10.7717/peerj.3747 (PMC5609522; doi:10.7717/peerj.3747)
Supplement: Table S1 [file peerj-05-3747-s003.pdf]

Transcription abundance of all BvAQP genes

Name	Gene code	Leaf	Taproot	Inflorescence	seeding
BvPIP1;1	Bv1g004510_fpur.t1	6.205204	324.368118	43.659561	4.113373
BvPIP1;2	Bv1g004520_shpz.t1	8.735936	271.5365755	56.967796	39.44222
BvPIP1;3	Bv2g024120_gqok.t1	481.184113	1411.468689	273.346741	842.2285
BvPIP2;1	Bv7g163390_cqnr.t1	92.918228	216.965729	147.662552	224.449
BvPIP2;2	Bv9g210030_ixem.t1	0	82.4510995	4.990615	290.3504
BvPIP2;3	Bv9g210020_yige.t1	0.926566	7.1934655	2.07546	15.79424
BvPIP2;4	Bv9g216070_reke.t1	79.946609	743.651337	291.931976	840.9092
BvTIP1;1	Bv7ug180930_kzkq.t1	1879.462402	1117.143189	194.240387	776.6156
BvTIP1;2	Bv2g037380_iuuk.t1	0	0.114297	12.075761	0.117749
BvTIP1;3	Bv7g176430_ynzf.t1	1.147823	92.6305275	4.847071	182.7608
BvTIP2;1	Bv9g223310_dkzm.t1	150.133667	128.0028765	61.597431	268.1113
BvTIP2;2	Bv5g104980_xunf.t1	0	47.7602215	0	108.4979
BvTIP3;1	Bv8g190600_dreg.t1	0	1.4354825	0.463279	4.340793
BvTIP4;1	Bv2g032200_ydno.t1	6.700949	371.3661345	64.998787	248.6495
BvTIP5;1	Bv3ug068240_gghp.t1	0	0	0.891779	0
BvNIP1;1	Bv8ug202570_ughi.t1	5.228934	2.719534	6.625377	16.20953
BvNIP4;1	Bv2g027680_aejh.t1	0	12.349268	2.269284	2.426967
BvNIP4;2	Bv2g027660_xash.t1	0	1.276335	1.277294	0.320927
BvNIP5;1	Bv6TE021760_gkiq.t1	0.07519	0.2982985	0.373979	0.092123
BvNIP5;2	Bv6g139140_oani.t1	11.497099	144.0580215	11.096544	63.44042
BvNIP6;1	Bv9g225280_hmzo.t1	27.709431	168.1454165	18.628855	7.763346
BvNIP6;2	Bv5g108450_zkgo.t1	0	0.608103	0.422167	3.233918
BvNIP6;3	Bv5g108440_jecw.t1	0	0	0	0
BvNIP7;1	Bv3ug070540_kqew.t1	0	0.3593385	5.749987	0.006317
BvSIP1;1α	Bv2g035790_zywx.t1	15.770978	11.248759	3.230541	5.463302
BvSIP1;1β	Bv2g035780_fzwq.t1	21.109791	23.3341825	16.814817	17.12506
BvSIP2;1	Bv3g064810_qzqg.t1	0.948852	2.182673	3.841118	22.51805
BvXIP1;1	Bv9g217040_iwpe.t1	8.606247	2.917488	1.587379	0.059568

Transcription abundance of all BvAQP genes

Name	seed	young leaf	young leaf (salt)	young leaf (heat)
BvPIP1;1	1.438739	63.114216	50.208286	41.594837
BvPIP1;2	15.007135	62.266872	43.013039	36.336815
BvPIP1;3	268.126465	481.735474	386.974701	766.257507
BvPIP2;1	319.232941	44.789177	36.631813	67.581169
BvPIP2;2	32.630814	1.019265	0.89456	6.006581
BvPIP2;3	0.167238	4.348359	1.856857	2.347586
BvPIP2;4	76.903748	386.642212	528.917114	749.330261
BvTIP1;1	108.438766	205.115555	862.178101	505.667999
BvTIP1;2	0	0	0	0
BvTIP1;3	11.160621	1.280572	0.885626	1.45266
BvTIP2;1	0.500361	108.375023	344.738281	449.011108
BvTIP2;2	0.983663	0.023529	0.030093	0.021434
BvTIP3;1	558.248901	0	0	0
BvTIP4;1	5.168621	20.825426	2.45968	25.147043
BvTIP5;1	0	0	0.006939	0
BvNIP1;1	6.030573	2.459336	0.817261	2.511876
BvNIP4;1	2.333961	2.269322	0.891222	0.602472
BvNIP4;2	1.495995	0.457919	0.121252	0.36433
BvNIP5;1	0	0.012337	0.002268	0.013157
BvNIP5;2	2.837413	6.64873	3.581954	3.264696
BvNIP6;1	1.760864	11.077249	5.330366	6.387152
BvNIP6;2	0	0	0	0
BvNIP6;3	0	0	0	0
BvNIP7;1	0	0.093104	0.049403	0.073512
BvSIP1;1α	3.412658	19.309324	9.570596	11.687196
BvSIP1;1β	9.522343	35.218609	19.464991	15.920908
BvSIP2;1	2.235126	36.36356	16.296434	17.156631
BvXIP1;1	0.699233	0.440801	0.389812	2.72492
